# Environmental controls on the detectability of hydrodynamic changes induced by offshore wind farm structures

**DOI:** 10.1038/s41598-026-71585-0

**Published:** 2026-10-07

**Authors:** Michela De Dominicis, Rory B. O’Hara Murray, Alejandro Gallego, Charlotte A. J. Williams, Beth E. Scott

**Affiliations:** 1 National Oceanography Centre, Liverpool, UK; 2 Scottish Government Marine Directorate, Aberdeen, UK; 3 University of Aberdeen, Aberdeen, UK

**Keywords:** Offshore wind energy, Wakes, Mixing, Stratification, Numerical modelling, Climate sciences, Environmental sciences, Ocean sciences

## Abstract

A combination of model simulations and field observations collected in 2023 and 2024 near Seagreen, Scotland’s largest Offshore Wind Farm (OWF), is used to assess when, where and by how much (physical) changes induced by OWFs structures become detectable and environmentally relevant. Seagreen has the deepest (> 55 m) fixed bottom foundations of any OWF in the world (in 2026), and in offshore deep (> 50 m) waters there is the potential for OWFs structures to perturb natural ocean mixing and modify seasonal stratification. We found that OWF impacts on local hydrodynamics are highly sensitive to environmental conditions, with the strongest effects occurring under weakly stratified conditions, in frontal/transitional zones, when the water column is most responsive to small increases in mixing. Summer average sea surface temperature decreases are generally between -0.1 and -0.3$^{\circ }$C within the farm and in its wake, but with cooling exceeding -0.5$^{\circ }$C during the 2023 marine heatwave. Due to high seasonal and monthly variability, OWF impact assessments based on limited observational periods may underestimate or overestimate the range of hydrodynamic changes. This work highlights the value of combining numerical models with observations: model simulations identified the periods and locations of detectable OWF-induced changes, while observations supported the modelled response.

## Introduction

The UK is committed to delivering up to 50 GW of offshore wind electricity generation capacity by 2030, including up to 5 GW of innovative floating wind^1^. There are still gaps in the understanding of the cumulative effects of a massive expansion of Offshore Wind Farms (OWFs) on marine life, especially in combination with other factors such as climate change and other human activities. The presence of the turbine underwater structures and the extraction of wind energy will perturb the natural physical environment, potentially causing cascading effects on other marine ecosystem components.

Existing OWFs have so far been generally installed in shallow coastal waters (< 50 m), usually tidally-mixed waters (except in regions of freshwater influence), where OWFs can enhance bed shear stress and mixing, with consequent effects on sediments distribution^2^. The expansion of OWFs is now moving into deeper waters (> 50 m) in the North Sea. These areas have lower tidal velocities and weaker natural tidal mixing than coastal waters, and they are typically seasonally stratified, when in summer the surface heating dominates over the mixing induced by tides. The installation of new OWFs in deeper stratified waters can potentially perturb the natural equilibrium of vertical mixing and stratification, with different impacts compared to coastal OWFs, which have not yet been fully investigated. OWFs could alter the exchange of water between the ocean bottom and the surface, which is crucial for the cycling of nutrients, oxygen and phytoplankton that form the base of the food chain supporting fish, seabirds and large marine mammals^3^.

**Fig. 1:**
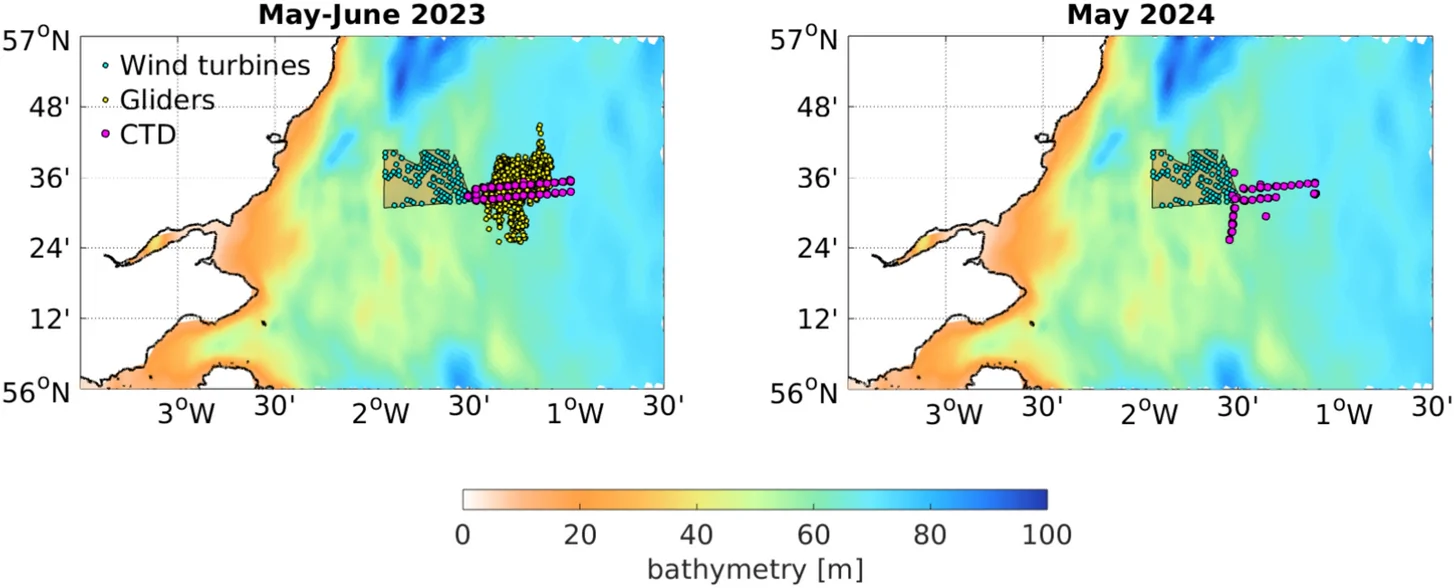
Locations of the Seagreen wind turbines, CTD and glider measurements in May-June 2023 and CTD deployments in May 2024.

Observations of physical changes due to the presence of OWFs are scarce. To date, only few studies have reported observations of OWF-induced mixing in occasionally stratified shallow waters (< 40 m). Floeter et al.^4^ reported a weakening of summer stratification in the vicinity of 80 wind turbines, not operational, thus the changes were due only to the presence of the underwater structures and were found to be within the natural variability observed in the area (German Bight, North Sea). Schultze et al.^5^ measured alterations in the vertical structure of the water column due to the presence of a single monopile, with changes depending on the level of stratification in the area. Hendriks et al.^6^ found a decrease of stratification in the wake of a single monopile in the Belgian part of the North Sea, with a well-defined band of colder, more saline water at the surface, and warmer, less saline water near the seabed. In this paper, we present and use for the first time observations taken in deep, seasonally stratified waters in the vicinity of the Seagreen wind farm, which has the deepest fixed bottom foundations (approximately at 59 m depth) of any OWF in the world. It was one of the largest construction projects ever undertaken in Scotland at the time of writing. The installation of the 114 jackets (steel frame structures) pinned to the seabed was completed in June 2023. Each structure supports a Vestas V164-10 MW turbine. The 1.1 GW Seagreen OWF is capable of generating enough electricity to power more than 1.6 million UK homes. Conductivity Temperature Depth (CTD) and glider data were collected next to the Seagreen OWF, as part of the PELAgIO project in 2023 and 2024 (see Fig. 1), described in Sect. “CTD and glider surveys”.

The mechanisms/pathways through which an OWF can change the mixing and stratification of the water column are: (i) structure drag and mixing, the induced drag generated by the underwater structures (fixed or floating underwater structures) and consequent induced mixing in the wake of the OWF; and (ii) wind wake, the modification of wind patterns in the wake of the OWF when it is operating. In this paper, we will focus on the potential physical changes induced by the drag and mixing generated by the presence of the underwater structures, but not the windwake. The majority of CTD and glider data collected in 2023 and 2024 were not within the wind wake (the wind was blowing in a different direction at the time of the observations), this means the effect of the underwater structures can be disentangled from the wind wake effect.

Numerical ocean model simulations are an important tool for assessing the potential impacts of OWFs that have not yet been built and for comparing the effects of different scales and layouts of development. These models also allow for comparison with the natural variability of the ocean system and with potential impacts from climate change. This is crucial for understanding the magnitude of the changes induced by OWFs and for defining acceptable levels of change. Therefore, it is essential to represent OWFs accurately in existing, widely used ocean models, which is the aim of this paper. Here, we use the Scottish Shelf Model (SSM), an FVCOM (Finite Volume Community Ocean Model)^7^ implementation covering the north-west European continental shelf, which is routinely used by the Marine Directorate of the Scottish Government^8–13^, further described in Sect. “The Scottish Shelf Model”.

Existing models evaluating underwater structure impacts can be divided into two categories: (i) models of relatively small spatial extent (1-2 kilometres) that are high-resolution non-hydrostatic models (usually micro-scale Large Eddy Simulations models) with individually resolved support towers or turbines, focused on effects local to turbine underwater structures within and close to individual OWFs; and (ii) models spanning much larger spatial extents (10s-100s kilometres) of lower resolution, such that individual turbines or OWFs are parameterised using (usually) sub-grid scale models and suited to capturing far-field effects across coastal and shelf regions. The sub-grid parameterisation of the underwater structure drag and wake turbulence described in Rennau et al.^14^, Carpenter et al.^15^, Christiansen et al.^16^ is based on an additional quadratic friction term in the momentum equations, as well as an additional production term in the dynamic equation for Turbulent Kinetic Energy (TKE) and its dissipation rate. Alternative methods have been used, for example Cazenave et al.^17^ used an unstructured grid where the wind monopiles are explicitly described in the grid, i.e. enabling representation of individual (cylindric) turbine underwater structures with no need for sub-grid specific parameterisation of structure-induced hydrodynamics and mixing. This approach has been further tested by Christiansen et al.^16^ and compared with the sub-grid parameterisation. With the grid resolving turbine underwater structures, Christiansen et al.^16^ encountered numerical problems, not previously identified by Cazenave et al.^17^, due to the high resolution and hydrostatic approximation. They instead recommend the sub-grid parameterisation as a suitable method for assessing the large-scale impacts of structure-induced mixing.

In this work, to reproduce the induced drag generated by the underwater structures of OWFs and the consequent induced mixing in the wake of the OWF, we integrated the sub-grid parameterisation approach in the FVCOM model and in the coupled General Ocean Turbulent Model (GOTM) (turbulence model^18^), following the method described in Sect. “The underwater structure drag and mixing parameterisation”. The results of the drag and mixing parameterisation are dependent on the choice of scaling parameters in the drag and mixing formulation, specifically the structure drag coefficient $C_{D}$ and the turbulence dissipation scaling parameter $c_{4}$, with the former introducing the greatest uncertainty. Our results show the seasonal and spatial variability of the structure-induced response, assess how environmental conditions influence its detectability, and compare modelled changes with CTD and glider observations. The seasonal and monthly maps of OWF-induced changes shown in this paper were obtained using a “medium” value of the drag coefficient ($C_{D}$ = 0.63, recommended state-of-the-art value^16^) and considering a strong mixing scenario ($c_{4}$= 0.6, suggested by Rennau et al.^14^). We also performed a total of 10 model simulations for spring and summer 2023 and 2024 to assess the uncertainty to $C_{D}$ and $c_{4}$ (see Supplementary Material, Table S1). One novelty of this paper is the comparison between observations and modelled changes to physical processes potentially caused by OWF underwater structures, shown in Sect. “Model-guided comparison with field observations” and in the Supplementary Material. This is necessary to build confidence in the model’s accuracy, something that is lacking in the current state-of-the-art literature.

## Results

### Seasonal variability of hydrodynamic changes induced by offshore wind farm structures

**Fig. 2:**
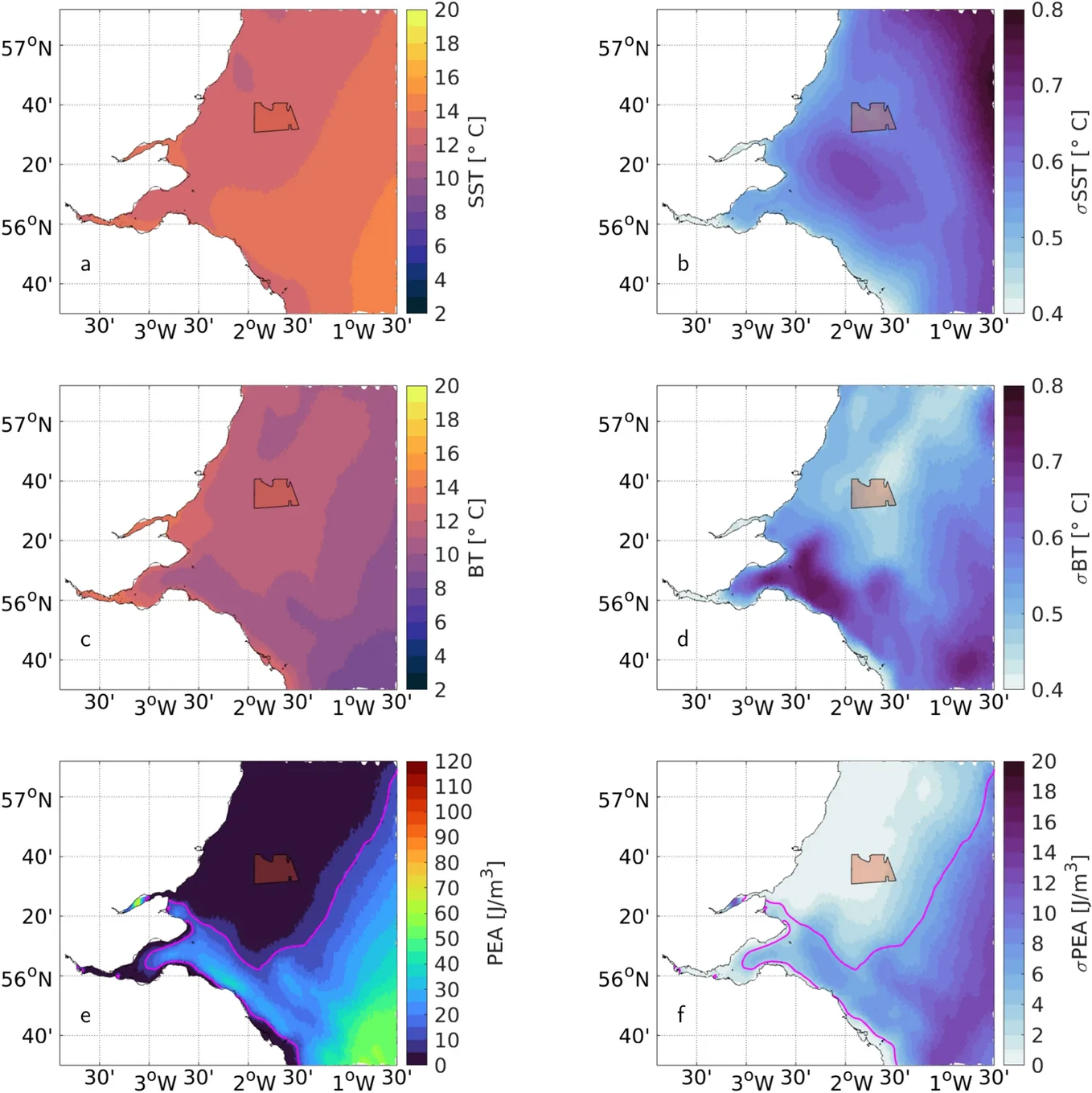
Summer (June-July-August - JJA) climatological average and variability for SST, BT and PEA calculated from the Scottish Shelf Waters Reanalysis Service 1993-2019run. The polygon outlines the perimeter of the Seagreen wind farm: (**a**) SST JJA 1993-2019 average; (**b**) SST JJA 1993-2019 standard deviation; (**c**) BT JJA 1993-2019 average; (**d**) BT JJA 1993-2019 standard deviation; (**e**) PEA JJA 1993-2019 average; (**f**) PEA JJA 1993-2019 standard deviation. In panel **e** and **f** the pink line is the PEA 10 J $\hbox {m}^{-3}$ contour separating mixed from stratified waters.

The location of the Seagreen wind farm and the summer (June-July-August; JJA) climatological average conditions for Sea Surface Temperature (SST), Bottom temperature (BT) and Potential Energy Anomaly (PEA) and their summer natural variability in the Firth of Forth (Scotland, UK) are shown in Fig. 2 (calculated from the 1993-2019 Scottish Shelf Model 3.02 - 27 Year Reanalysis^12,13^). PEA is defined as the amount of energy required to fully mix the water column^19^. Typical thresholds separating mixed from seasonally stratified waters are of the order of 10-20 J m^-3^, waters on-shore of the 10 J m^-3^ contour tends to be well mixed^20^. Whilst the Seagreen OWF is on average within the summer (JJA) mixed region (see summer climatological PEA in Fig. 2e), it can become stratified for shorter periods of time during the summer.

**Fig. 3:**
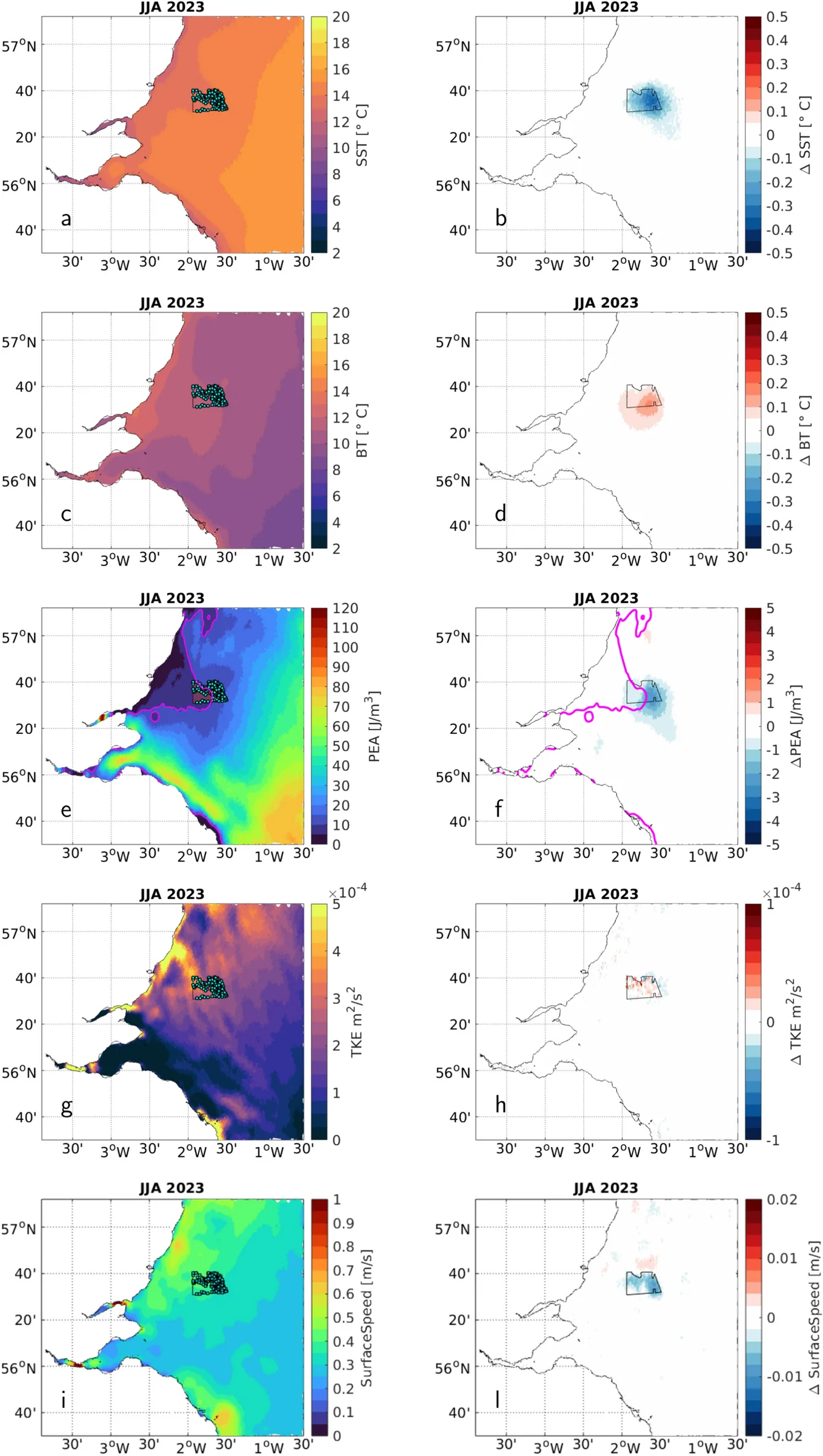
Summer (June-July-August JJA) 2023 seasonal changes due to the Seagreen wind farm structure wake ($C_{D}=0.63$, $c_{4}=0.6$). Changes are calculated as the difference between the seasonal average simulation including the OWF and the baseline simulation without the OWF. Panels show: (**a**) SST JJA 2023; (**b**) changes SST JJA 2023; (**c**) BT JJA 2023; (**d**) changes BT JJA 2023; (**e**) PEA JJA 2023; (**f**) changes PEA JJA 2023, the pink line is the 10 J/$\hbox {m}^{-3}$ contour representing the limit between mixed and stratified waters; (**g**) mid-water column TKE JJA 2023; (**h**) changes mid-water column TKE JJA 2023; (**i**) surface current speed JJA 2023; (**l**) changes surface current speed JJA 2023. The polygon outlines the perimeter of the Seagreen wind farm and the light blue dots are the locations of the wind turbines.

In this paper, we present the results of the spring and summer 2023 and 2024 model simulations, reproducing ocean conditions with/without the Seagreen wind farm, in terms of summer (JJA) seasonal and monthly (May, June, July, August) average changes, due the presence of the Seagreen wind farm, to SST, BT, PEA, TKE and current speed. Figure 3 shows the summer (JJA) 2023 averages of SST (Fig. 3a) and BT (Fig. 3c), along with the changes associated with the presence of the Seagreen wind farm. The SST exhibits a decrease that can reach up to -0.3$^{\circ }$C on average for the season (Fig. 3b), while the BT shows an increase of up to 0.15$^{\circ }$C (Fig. 3d). These changes are below the natural summer (JJA) variability in the area, which is in the range 0.5-0.6$^{\circ }$C for SST (see Fig. 2b) and 0.4-0.5$^{\circ }$C for BT (see Fig. 2d). The opposing trends (SST decrease and BT increase) indicate that surface and bottom waters are becoming more similar in temperature, which corresponds to a reduction in water column stratification. 2023 has higher summer (JJA) PEA (Fig. 3e) than the climatological average (Fig. 2e), and the Seagreen OWF is located within the transition zone between mixed and seasonally stratified waters, identified by the 10 J m^-3^ contour line (Fig. 3e). A more uniform vertical thermal structure, with surface waters cooling (SST decrease) and bottom waters warming (BT increase), and therefore a more uniform density, means a decrease in PEA, with reductions reaching up to approximately -3 J m^-3^. This change is of the same order of magnitude of the natural variability of PEA in the region (see Fig. 2f), however, unlike the natural variability, which can act in either direction, the OWF effect is consistently unidirectional, resulting in enhanced mixing. Thus, although the magnitude of the response is similar to the natural variability, its persistent direction distinguishes the OWF-induced change from natural fluctuations. The primary driver of the stratification changes is the increase in TKE, in Fig. 3h we show the mid-water column TKE increase, which is confined to the area occupied by the OWF. The increase in TKE is due to the transformation into turbulence of the lost of kinetic energy due to the deceleration generated by the drag of underwater structure (see Sect. “The underwater structure drag and mixing parameterisation”). The signal of the water deceleration is visible in the reduction of the current velocities at the surface. The summer (JJA) average of the surface current speed during 2023 (Fig. 3i) shows the Seagreen wind farm is in area of relatively high surface current speed (on average 0.4-0.5 m/s), which are reduced by up to 0.01 m/s (about 3%) within the Seagreen wind farm by the drag of the OWF underwater structures (Fig. 3l). We found that the drag exerted by the underwater structures reduces surface current speeds, but this effect is confined to the upper water column and is accompanied by increased mid-depth current speed throughout both years (see Supplementary Material).

**Fig. 4:**
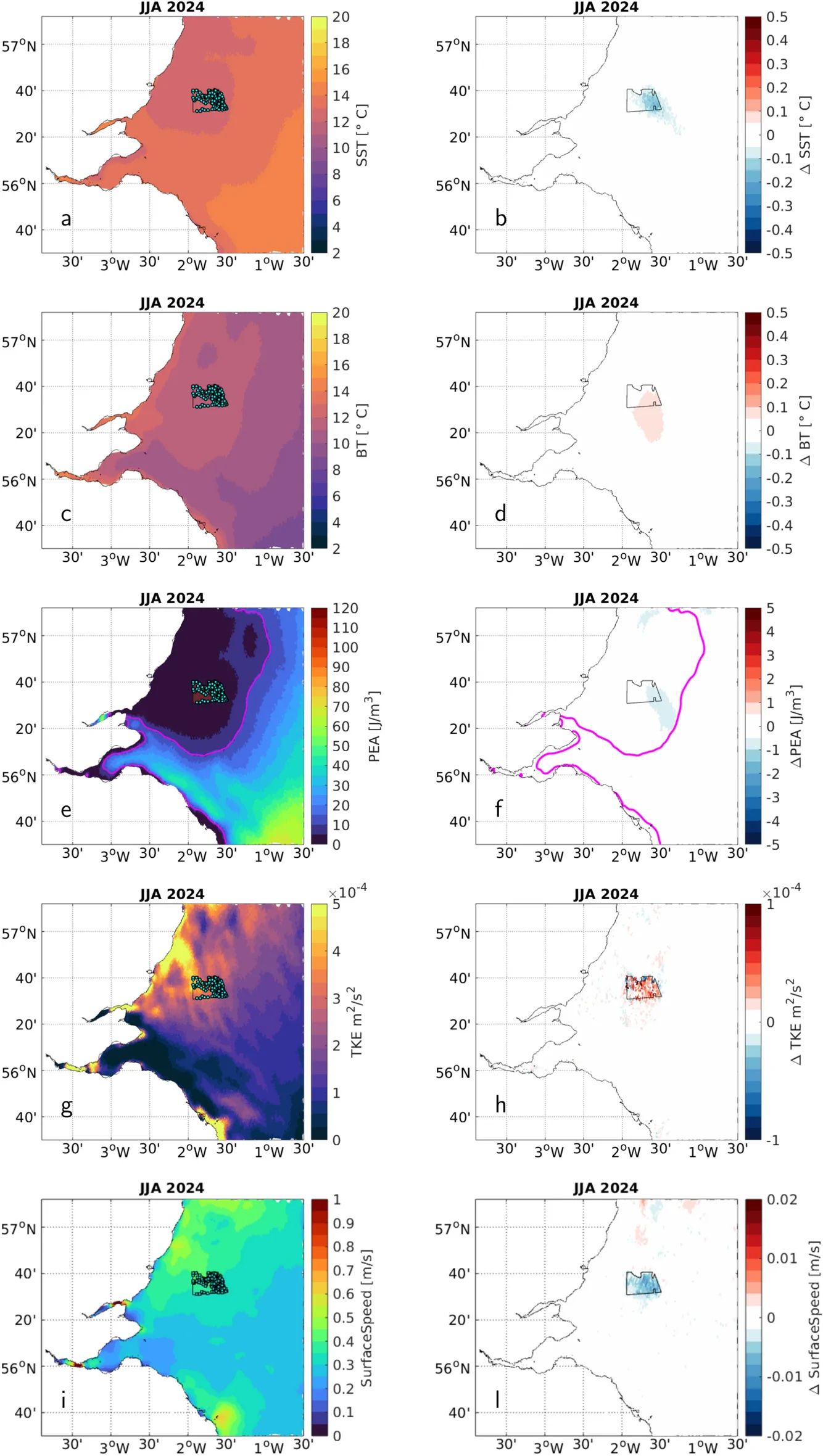
Summer (June-July-August JJA) 2024 seasonal changes due to the Seagreen wind farm structure wake ($C_{D}=0.63$, $c_{4}=0.6$). Changes are calculated as the difference between the seasonal average simulation including the OWF and the baseline simulation without the OWF: (**a**) SST JJA 2024; (**b**) changes SST JJA 2024; (**c**) BT JJA 2024; (**d**) changes BT JJA 2024; (**e**) PEA JJA 2024; (**f**) changes PEA JJA 2024, the pink line is the 10 J/$\hbox {m}^3$ contour representing the limit between mixed and stratified waters; (**g**) mid-water column TKE JJA 2024; (**h**) changes mid-water column TKE JJA 2024; (**i**) surface current speed JJA 2024; (**l**) changes surface current speed JJA 2024. The polygon outlines the perimeter of the Seagreen wind farm and the light blue dots are the locations of the wind turbines.

In 2024, summer seasonal average changes display a similar spatial pattern to those in 2023, though with reduced magnitude (Fig. 4). SST decreases by up to -0.2$^{\circ }$C (Fig. 4b), while BT increases by up to 0.1$^{\circ }$C (Fig. 4d). The 2024 PEA field (Fig. 4e) resembles that of the climatological average (Fig. 2e), with the OWF within the mixed region on average. Stratification changes are minimal, with PEA reductions of only up to about -1 J m^-3^ (Fig. 4f). Thus, despite the fact that mid-water column TKE increases are larger in 2024 than in 2023 (Fig. 4h), they do not influence stratification simply because the water column was already mixed. In 2024, the summer seasonal average of the surface current speed changes (Fig. 4l) show similar magnitude to 2023 (Fig. 3l). Overall, stratification changes in 2024 are much weaker than in 2023, and well below the natural variability in the region (see Fig. 2f). This is largely because 2023 was an anomalous year characterized by a strong marine heatwave^21^, which produced a more extensive region of seasonal stratification.  

In summary, the response of the water column to the Seagreen wind farm is strongly modulated by the background stratification state. In 2023, when a marine heatwave produced unusually strong and widespread stratification, OWF-induced mixing was able to erode the stratification, leading to substantial reductions in both SST and PEA. In contrast, in 2024 the water column around the OWF was largely mixed, meaning that even though the increase in TKE is greater in 2024 than 2023, its effect on temperature structure and stratification is negligible due to the absence of stratified conditions. These results demonstrate that the impact of OWFs on local hydrodynamics is highly sensitive to environmental context, with the strongest effects occurring in transitional/frontal waters when the water column is already weakly stratified and therefore more responsive to marginal increases in mixing.

### Model-guided comparison with field observations

Field work was carried out in 2023 and 2024 in the vicinity of the Seagreen OWF to characterise the physical and biological environment and to acquire data to calibrate and validate numerical models, as part of the PELAgIO project in 2023 and 2024. The work involved the deployment of autonomous gliders and ship-based activities including the deployment of landers (with acoustic current profilers, echosounders and CTD instruments), profiling CTDs (and associated sensors for dissolved oxygen and chlorophyll fluorescence) with water sampling for biogeochemical measurements and sensor calibration, acoustic transects using a fisheries echosounder (EK60) and direct sea bird observations in some of the ship surveys (spring).

**Fig. 5:**
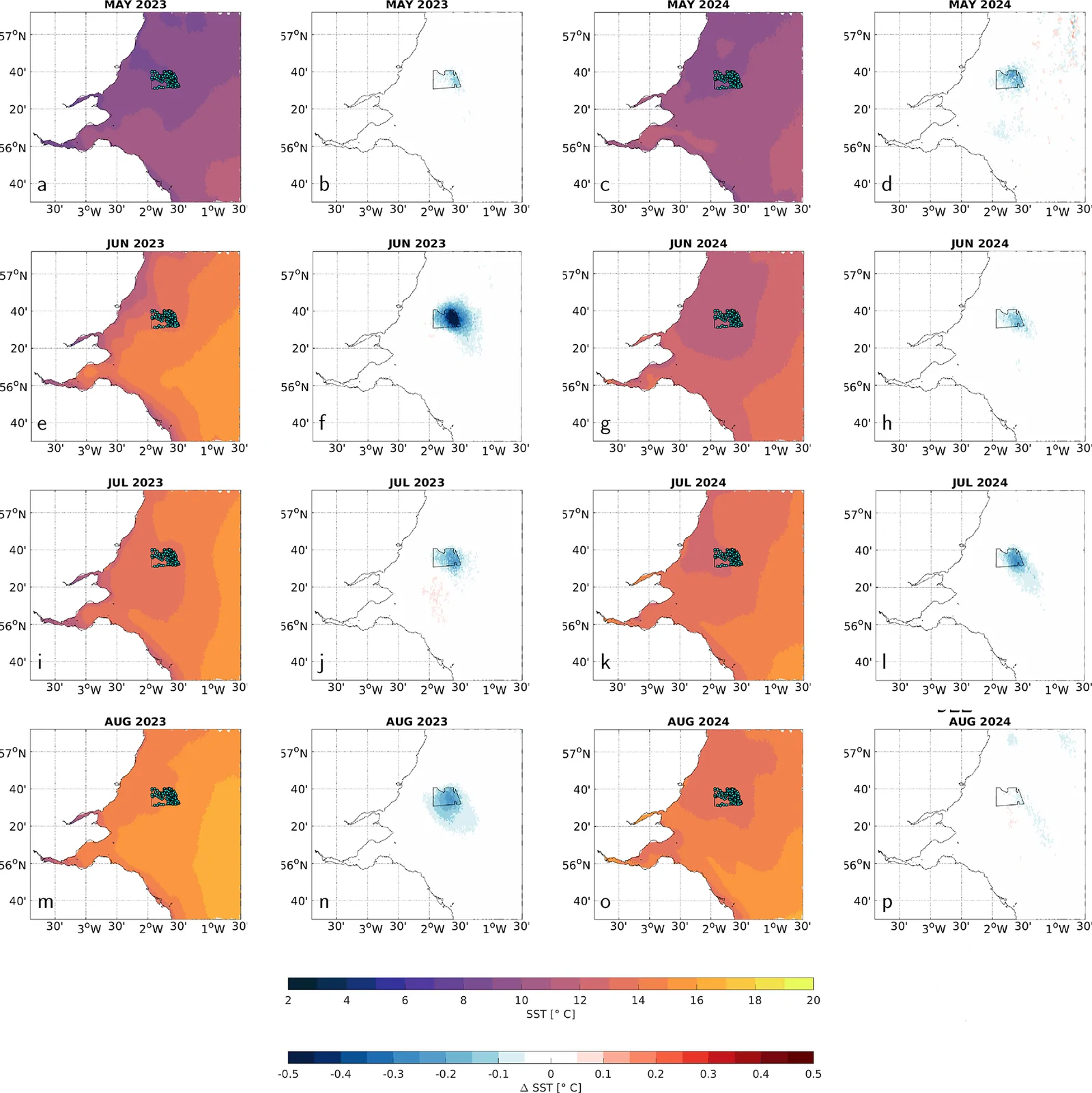
Monthly average SST and changes due to the Seagreen wind farm structure wake in 2023 and 2024 ($C_{D}=0.63$, $c_{4}=0.6$). SST changes are calculated as the difference between the monthly average simulation including the OWF and the baseline simulation without the OWF. Panels show: (**a**) SST May 2023; (**b**) SST changes May 2023; (**c**) SST May 2024; (**d**) SST changes May 2024; (**e**) SST June 2023; (**f**) SST changes June 2023; (**g**) SST June 2024; (**h**) SST changes June 2024; (**i**) SST July 2023; (**j**) SST changes July 2023; (**k**) SST July 2024; (**l**) SST changes July 2024; (**m**) SST August 2023; (**n**) SST changes August 2023; (**o**) SST August 2024; (**p**) SST changes August 2024. The polygon outlines the perimeter of the Seagreen wind farm and the light blue dots are the locations of the wind turbines.

To understand when and where OWF-induced temperature changes could be detected by CTDs and gliders, we analysed modelled monthly changes in SST and BT and used these results to guide the selection of the glider and ship-based CTD observations to be compared with model simulations (see Fig. 1). CTD data were collected in May 2023 and May 2024 (see Sect. “CTD and glider surveys”). Examining the summer changes as monthly averages reveals that May typically exhibits smaller or negligible changes in SST (Fig. 5b,d), BT (see Supplementary Material, Fig. S1b,d), and PEA (see Supplementary Material, Fig. S3b,d). The largest reductions, exceeding 0.02 m/s, in surface current speed occurred in May 2023 and May 2024 (see Supplementary Material, Fig. S4b,d). Consequently, changes in TKE (see Supplementary Material, Fig. S2b,d) are larger in May in both years than in any summer month, however their effect on stratification is negligible because the waters at the Seagreen wind farm location are not tipically stratified during May (see Supplementary Material, Fig. S3a,c). This means that detectability of OWF-induced temperature changes using the CTD data collected in May 2023 and 2024 is limited. Overall, during the CTD survey periods (May 2023 and May 2024) the OWF-induced temperature changes, the sensitivity to the model parameters influencing the structure wake (drag coefficient, $C_{D}$, and turbulence dissipation scaling parameter, $c_{4}$, see Sect. “The underwater structure drag and mixing parameterisation”), and the model error are approximately *±*0.1-0.2$^{\circ }$C (see Supplementary Material). In general, larger changes would be expected later in the summer, a more stratified period, and in locations where the largest temperature changes are modelled to occur, primarily within the OWF itself, rather than at the locations where the CTD measurements were collected (CTD collections and glider routes were limited to locations outside the windfarm due to ongoing construction activities).  

Unlike the May CTD surveys, the gliders sampled during a period of strong stratification and during the peak of the 2023 marine heatwave, when model simulations indicate that OWF-induced temperature changes were at their maximum. OWF-induced changes in SST reach their maximum in June 2023 (Fig. 5f), with negative anomalies exceeding -0.5$^{\circ }$C. This occurs because SST and stratification were anomalously high in June 2023 due to the marine heatwave occurring at the time^21^: the enhanced surface warming increased the vertical temperature gradient, strengthening stratification and further suppressing vertical mixing between the surface and deeper waters. During the other summer months in both 2023 and 2024 (Fig. 5j,l,n,p), the OWF-induced SST changes are consistently around -0.1 to -0.2$^{\circ }$C, except for August 2024 (Fig. 5p), when the changes are negligible. Notably, there are no detectable SST changes in August 2024 despite relatively large TKE anomalies (see Supplementary Material, Fig. S2p). BT variability in 2023 and 2024 is much smaller than the variability observed at the surface, leading to a more uniform response in BT compared to SST. OWF-induced changes in BT remain consistently around 0.1-0.15$^{\circ }$C (see Supplementary Material, Fig. S1), reaching peaks of 0.2$^{\circ }$C in 2023 only (see Supplementary Material, Fig. S1f,n).

Thanks to the model simulations, we were able to identify the period when the gliders were most likely located within the areas affected by the OWF structures and when the OWF-induced changes were largest (see Fig. 5f). This would have been difficult to determine using observations alone without prior guidance from the model simulations. The relevant period corresponds to the approximately 10 days of the June 2023 marine heatwave, between 12 and 22 June. During this period, the gliders collected more than 1000 profiles, typically covering from 10 m above the seabed to 5 m below the surface. However, only 64 profiles reached the sea surface, where the largest temperature changes are predicted. A mean RMSE of approximately 0.93$^\circ$C across the 64 profiles is obtained. This is larger than the RMSE obtained from the CTD observations (see Supplementary Material), with the model error showing an increasing trend through the summer. A consistent negative surface temperature bias of -0.20 to -0.46$^\circ$C was found across the simulations, while the bottom temperature bias was consistently positive and substantially larger (0.84$^\circ$C). The large bottom temperature bias indicates that the model systematically overestimates temperatures at the deepest glider observations, contributing to the higher RMSE compared with the CTD profiles. This larger discrepancy likely reflects the seasonal evolution of the water column, as stratification and associated temperature gradients become stronger during summer.

**Fig. 6:**
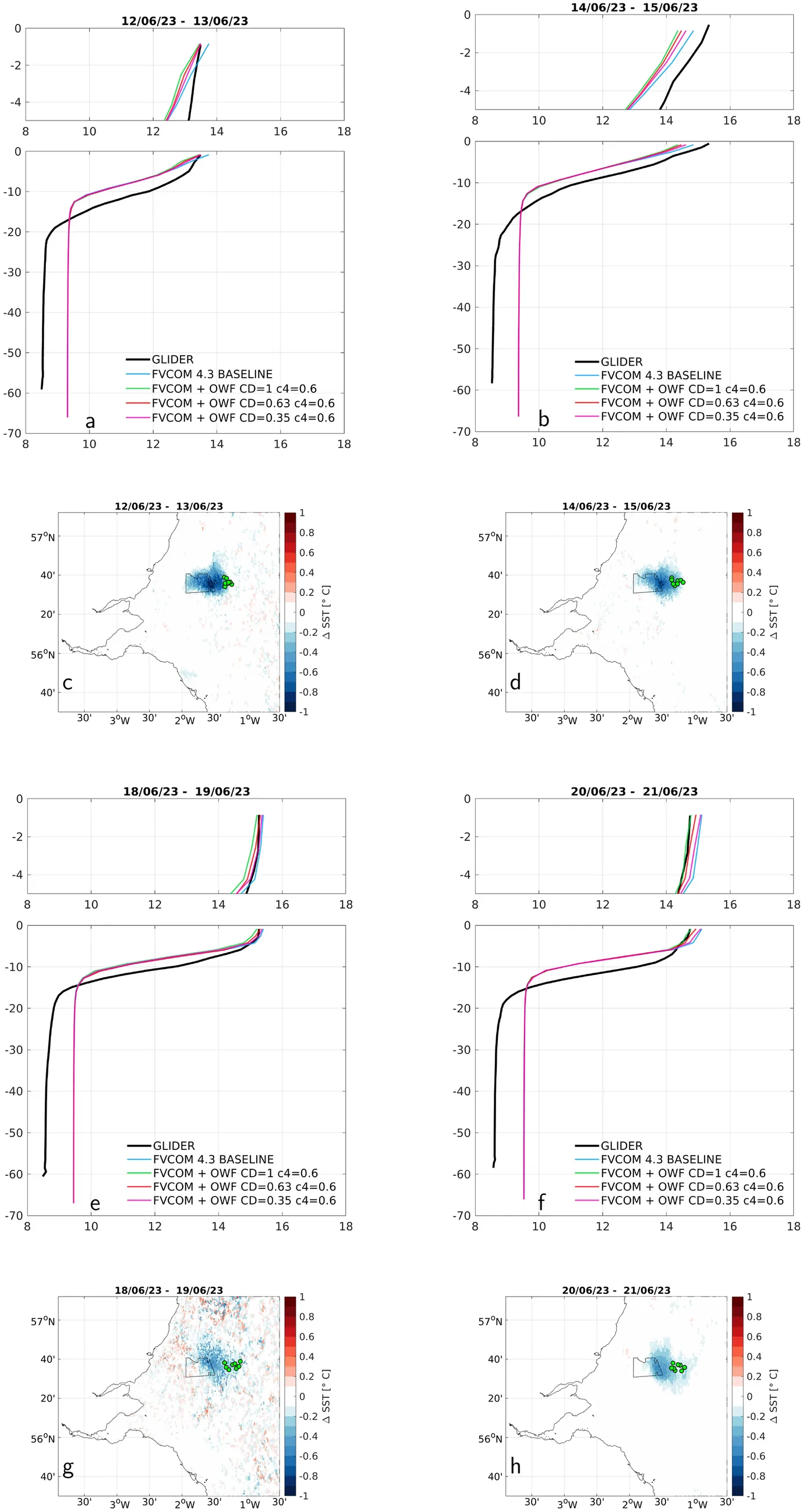
Comparison of glider temperature profiles collected within the OWF wake (black) and model simulations (coloured lines) between 12 and 22 June 2023. Glider profiles were averaged over clusters of eight consecutive casts, and only the average of those collected while the glider was located within the OWF wake are shown. Panel (**a**), (**b**), (**e**), and (**f**) compare the averaged glider temperature profile with simulated temperature profiles for different values of the drag coefficient $C_{D}$ (with $c_{4}=0.6$). Panels (**c**), (**d**), (**g**), and (**h**) also show the corresponding modelled SST changes for $C_{D}=0.63$, used to identify periods when the glider was within the structure wake of the Seagreen wind farm.

In Fig. 6, we show average glider temperature profiles, computed over a cluster of 8 consecutive profiles, compared with simulated temperature profiles for different drag coefficient values ($C_{D}$) to give an indication of model uncertainty (see Sect. “The underwater structure drag and mixing parameterisation”). To highlight the OWF’s effect, we also show the average SST changes calculated for $C_{D}$ = 0.63 over the same period, which enabled us to identify when the gliders were located within the areas affected by the OWF structures (see Fig. 6c,d,g,h). Only the averaged profiles corresponding to times within the structure wake are plotted.

The SST changes associated with the presence of the Seagreen wind farm reached their maximum between 12 and 15 June 2023 (Fig. 6c,d). On 12 June 2023, SST reductions exceeded -1$^{\circ }$C within the OWF area (Fig. 6c), which is larger than the natural variability of the system. However, these large anomalies persisted for less than one week and occurred under exceptional environmental conditions associated with the marine heatwave. Although the SST changes in June 2023 are larger than those observed during May 2023, the sensitivity to the drag coefficient remains limited. On 12 June 2023, the SST change induced by the OWF at the location of the gliders, averaged over 8 profiles (approximately a 24-hour period), is about -0.2$^{\circ }$C, with only small differences between simulations using different $C_D$ values (Fig. 6a). The simulations including the OWF reproduce the observed surface temperature measured by the glider better than the baseline simulation without the OWF (blue line in Fig. 6a). This agreement provides confidence in the modelled SST changes. While this does not demonstrate a direct observational detection of the OWF signal, it suggests that the inclusion of OWF-induced mixing improves the representation of surface temperature during this period.

The glider entered the structure wake again on 14-15 June 2023 (Fig. 6d). In this case, the model underestimates the observed SST by approximately 0.5$^{\circ }$C (Fig. 6b). The sensitivity to the tested $C_D$ values spans about 0.3$^{\circ }$C, and the mismatch with the observations would increase to approximately -1$^{\circ }$C when using the largest $C_D$ value. During 18-19 June 2023, the agreement between the modelled and observed OWF is very good: the modelled temperature profile with $C_D$ = 0.63 better matches the observed temperature profile (in the top few meters, red line in Fig. 6e). The sensitivity to $C_D$ is in the range of 0.3$^{\circ }$C. In contrast, on 20-21 June 2023, the simulation with $C_D$ = 1 (green line Fig. 6f) reproduces the observed SST almost perfectly. In this case, the SST reduction induced by the OWF is about -0.3$^{\circ }$C, and the spread among simulations using different $C_D$ values is also approximately 0.3$^{\circ }$C.    

The overall conclusions is that OWF-induced surface temperature changes, the sensitivity to model parameters ($C_{D}$) are all approximately *±*0.2-0.3$^{\circ }$C, which is below the model inherent error. The limited sensitivity can partly be attributed to the fact that the measurements were not taken within the OWF itself. In addition, because the drag-induced retarding force depends on both $C_{D}$ and the tidal velocities, a stronger sensitivity would be expected in more tidally energetic environments. Overall, these results suggest that using mid-range drag coefficient values provides realistic simulations of the OWF-induced changes, with an uncertainty of approximately *±*0.2-0.3$^{\circ }$C.

## Discussion

Overall, our results demonstrate that the hydrodynamic response to the presence of the Seagreen wind farm is strongly conditioned by the background stratification regime. While structure-induced turbulence consistently enhances TKE within the OWF footprint, its impact on the thermal structure remains modest, with average summer seasonal SST reductions generally of the order of -0.1 to -0.3$^{\circ }$C, BT increases of around 0.1-0.15$^{\circ }$C, and PEA decreases typically between -1 and -3 J m^-3^, reaching about -4 J m during stronger stratified periods. These changes are of a similar magnitude to the natural variability observed across the region, however, the structure-induced response is consistently directed towards enhanced mixing, whereas the natural variability can produce changes in either direction. This magnitude of change is broadly in agreement with the most recent state of the art literature. While there is variability in environmental conditions (in both space and time) and magnitude of changes, most recent modelling studies show similar TKE spatial patterns and similar order of magnitude reduction of SST, PEA^16,22–24^. A recent observational study^6^, also reports similar processes and patterns, showing a near-surface water temperature decrease, near-bed temperature increase, and a decrease in stratification.

We found that drag exerted by the underwater structures reduces surface current speeds, with the maximum reduction during May 2023 and May 2024 reaching up to 0.02 m/s (5%), consistent with Christiansen et al.^22^ results. Similar to the observations reported by Hendriks et al.^6^, the reduction in current speed is primarily confined to the upper water column, with current speed changes detectable down to mid-depth, whereas the wake is less discernible near the seabed. This vertical structure explains why changes in depth-averaged current speed reported by Christiansen et al.^16^ were an order of magnitude smaller, as the current reduction is largely confined to the upper part of the water column. Interestingly, and to our knowledge not previously reported in other studies, we found that the reduction in current speed in the upper water column is accompanied by an increase at mid-depth (see Supplementary Material).

Monthly analysis further indicates that, during May, corresponding to the PELAgIO CTD observational survey in 2023 and 2024, SST, BT, and PEA responses were small or negligible because stratification was not yet developed, despite relatively large increases in TKE. The May CTD measurements therefore likely captured the lower bound of seasonal impacts. OWF-induced changes are larger when stratification is stronger, during summer months and in particular in June 2023, which was an extremely hot month, with changes in the average monthly SST exceeding 0.5$^{\circ }$C, which is however still within the natural variability in the area.

The comparison between model simulations and observations (CTD data in May 2023 and 2024 and glider data in June 2023) shows that the surface temperature changes induced by the OWF underwater structures are present but are below the model error. Importantly, these observations represent the first and, to date, only in situ measurements of structure-induced hydrodynamic effects at an OWF site in UK waters, highlighting both their novelty and the scarcity of observational constraints. Future campaigns would benefit from targeting periods of stronger stratification, when model results suggest the largest responses, and from sampling closer to turbine structures and along the direction of the structure wake, where effects are expected to be strongest.

Observational evidence of hydrodynamic impacts from operational OWFs remains scarce, and to our knowledge this study represents one of the first efforts to evaluate such effects using in situ observations in combination with modelling. These results therefore provide an initial observationally informed assessment, while also highlighting the need for more extensive, well planned and sustained measurement programmes. By combining observational data with numerical models, it was possible to extend the assessment beyond the measurement period and evaluate seasonal and interannual variability of the OWF-induced effects. OWF underwater structures reduce surface current speed, enhance ocean vertical mixing, reduce sea surface temperature and increase bottom temperature. The impact magnitude is highly context-dependent, with stratification strength playing a critical role. Assessments based on limited observation periods may underestimate or overestimate the potential range of the OWF-induced variability.

Numerical modelling plays a central role in this assessment. Observational datasets are typically limited in spatial and temporal coverage and cannot be used to characterise conditions prior to an OWF installation or operation. Model simulations provide an essential and affordable tool for evaluating potential impacts and exploring the sensitivity to different OWF locations and size. However, robust site-specific and regional observations collected near operational OWFs remain crucial for model calibration and validation. In turn, model results should guide the optimisation of observational strategies at future OWF sites, ensuring that monitoring efforts target the most informative locations and time periods, where OWF-induced changes are detectable. For example, as we presented in the paper, May 2023 was not the best period for the CTD deployments at this location, in order to quantify the OWF impact on temperature structure, just shifting the survey by one month would have allowed us to get a more robust dataset. However, we acknowledge that the ideal timing (or even location) of an observational programme may not be fully compatible with operational considerations. Also, we found that changes tend to be localised, at least for an OWF the size of Seagreen, and observations should be taken closer or inside the OWFs and within the modelled wake of the structure. This degree of sampling precision requires good logistical coordination and close agreement between scientific studies and offshore renewable energy industries.

In general, the results of the parameterisation of the underwater structure drag and wake turbulence are dependent on the choice of scaling parameters. We assumed that $\hbox {C}_D$ is constant, as done by Rennau et al.^14^, Carpenter et al.^15^ and Christiansen et al.^16^: in reality it is variable and dependent on the Reynolds number, surface roughness and structure geometry. The drag is also likely a function of depth and time and will vary with background shear and Froude number^25^. Magnitude of changes however are still uncertain due to the uncertainty associated with turbulent mixing processes in stratified waters^25^ and the mixing schemes used in ocean models can bias the effects of stratification^26^. In the present study, the simulated OWF changes induced by the Seagreen wind farm underwater structures during the measurement periods and the sensitivity to the drag coefficient ($C_D$) over the tested range (0.35-1) are detectable but remain within an uncertainty of approximately *±*0.2-0.3$^\circ$C. The drag-induced retarding force depends on both $C_D$ and tidal velocity; therefore, a stronger sensitivity is expected in more tidally energetic environments, where drag-induced mixing is enhanced^23^. Although stronger tidal currents would produce a larger deceleration, they may not lead to larger changes in the temperature profile, as the increased background tidal mixing would reduce the water-column stratification. Overall, we believe the magnitude of the hydrodynamic changes generated by the OWF fixed underwater structures are within the range simulated in this paper. Otherwise, a larger discrepancy would have been observed between the in-situ observations (2023-2024) and modelled profiles.

The expected magnitude of the structure-induced effects of offshore fixed wind infrastructure at the current construction levels is emerging. While this work focuses on the Seagreen wind farm, we recommend that these processes are considered at a regional scale for the cumulative impact assessment of future planned OWFs. This paper highlights the value of numerical models, when suitably validated with field observations collected at the appropriate spatial and temporal scales, for assessing and monitoring the potential effects of offshore wind developments on Good Environmental Status (GES), a central aim of the EU Marine Strategy Framework Directive. This is particularly relevant for Descriptor 7 (“hydrographic conditions”), which may be threatened for the first time by the large-scale expansion of offshore wind in seasonally stratified shelf seas.

Regional and shelf wide numerical models are fundamental for understanding the potential cumulative environmental impact of multiple developments once observations have been collected across a range of environmental settings. In this context, modelling may be most impactful during early marine spatial planning phases, when broad scale area selection decisions are made, as it can help identify locations with the smallest environmental impact for OWF deployment while accounting for cumulative interactions among multiple developments. Similarly, this work can be used to inform marine planning and policy, where decisions are made balancing the potential environmental impact with other factors such as economic development and energy supply aspirations. In contrast, modelling applied at the Environmental Impact Assessment stage for individual OWFs will have limited ability to influence outcomes at a regional scale, as the location of wind farm is already fixed. There is, however, some scope for potentially higher resolution modelling to explore how the micro-siting of individual turbines could affect the local oceanography of the area. Because OWF impacts on local hydrodynamics are highly sensitive to the surrounding environmental context, the choice of site often has a far greater influence on the resulting changes than the specific layout or turbine configuration of a wind farm.

In this work we focused on the structure wake, however, the magnitude of the changes induced by the wind wake can be larger than the magnitude of the structure wake, as recently reported by Christiansen et al.^22^ using an empirical wind wake parameterisation. Future work should incorporate atmospheric models or fully coupled atmosphere-ocean modelling systems in order to better represent feedbacks between offshore wind turbine atmospheric wakes, ocean mixing, and stratification. OWFs modify not only ocean dynamics through structure-induced turbulence but also the atmospheric boundary layer through wake generation, changes in momentum flux, and altered heat exchange at the sea surface. These atmospheric responses can in turn influence wind stress, surface fluxes, and mixing processes in the ocean, creating two-way interactions that are not captured in ocean-only simulations. Accounting for such coupled dynamics would allow a more complete representation of how OWFs modify both atmospheric and oceanic boundary layers across temporal and spatial scales.

The present work focuses exclusively on hydrodynamic effects. Future studies will extend this approach to include biogeochemical responses. If OWFs have the potential to perturb the natural balance between mixing and stratification, this can lead to potential alterations of the distribution of nutrients, oxygen, and phytoplankton (Jardine et al, in preparation). Such physical and biogeochemical changes may cascade through the ecosystem, affecting habitat structure, biodiversity, fish populations, seabirds, and marine mammals (Trifonova et al, in preparation). Additionally, these results highlight that OWFs may provide a mechanism to distribute heat into the ocean interior during severe surface marine heatwaves, such as the category 4 (most severe) marine heat wave in June 2023, potentially impacting biogeochemistry below the thermocline.

Finally, the ecological and climatic implications of enhanced mixing remain ambiguous. Increased vertical mixing may in some contexts alleviate excessive stratification and has been discussed as a potential nature-based contribution to climate resilience^25^, yet it may also alter nutrient fluxes, ecosystem structure, or heat distribution in ways that are not universally beneficial. Moreover, the wind wake might have the opposing effect of decreasing vertical mixing^22^. Future studies will explicitly account for both the wind wake and structure wake in the context of climate change, which is expected to modify background stratification and mixing regimes and may interact synergistically with OWF effects (Jardine et al, in preparation). Future work should be focused on the understanding of the physical and biogeochemical changes, to define acceptable thresholds of change. It will require placing OWF-induced variability not only in the context of natural variability, but also of the longer-term climate-driven trends.

## Method

### CTD and glider surveys

In 2023, CTD profiles were carried out along two 30 km parallel (spacing 3.5 km) approximately east-west (85$^{\circ }$ from north), onshore-offshore transects which cut across the interface between mixed and seasonally stratified waters, which was also the gliders area of operation and downstream of the prevailing wind wake of the OWF (the closest CTD station to Seagreen was 2 km to the east of the nearest turbine). The ships used for these deployments were RV Corystes (May 2023) and MRV Scotia (September and December 2023). A total of 126 CTD profiles were acquired in 2023.

In 2024, CTD profiles were carried out along a shorter version (< 23 km) of the same east-west transects but also along a 19 km, approximately north-south transect (189$^{\circ }$ from north) to the south of the OWF, aligned with the structure wake (the closest CTD station on that transect was 2 km from the nearest turbine). All CTD deployments in 2024 were carried out from MRV Scotia (May, September-October and December). A total of 134 CTD profiles were acquired in 2024. In this work we used only a selection of the CTD data: 89 profiles collected in May 2023 and 77 profiles collected in May 2024 (pinks dots, see Fig. 1).

A NOC shallow-water-rated, buoyancy driven Teledyne Webb Slocum glider (unit 398) was deployed on 26 April 2023 from the MV Temora. The glider was equipped with a Seabird SBE42 GPCTD sensor to measure temperature, conductivity and pressure. The glider was operated and piloted by MARS (Marine Autonomous Robotic Systems at the National Oceanography Centre, Southampton) to maintain repetitions of a 16 km long E-W transect between two waypoints (56.617 N, 1.375 W and 56.642 N, 1.117 W) until 10 August 2023, when it was recovered by the MPV Minna. The glider collected measurements within 10 m of the seabed and 5 m of the surface on most dives, but reaching the sea surface every 2-3 hrs, with each dive taking approximately 20 minutes. Each leg of the transect took < 1 day to complete. Glider conductivity was corrected for thermal inertia, and nearby (< 1 km) CTD measurements collected by the RV Corystes (5-10 May 2023) were used to calibrate the derived glider salinity. Sea water density was subsequently calculated from temperature, salinity and pressure. In this work we used only the data collected in June 2023 (yellow dots, see Fig. 1).

### The Scottish Shelf Model

In this work we use the Scottish Shelf Model (SSM) which has been in iterative development since 2012^8–13^. Its strength is the use of an unstructured grid focusing higher resolution cells on coastal areas based on FVCOM. The domain covers most of UK waters, the North Sea and the English Channel, with the Scottish continental shelf at relatively high resolution up to 0.5 km close to the coast and at a resolution of 1.5-2km where the Seagreen wind farm is located^9,11^. Here, we use a similar model setup to the Scottish Shelf Model (SSM) 3.02 - 27 Year Reanalysis^12,13^, otherwise referred to as the Scottish Shelf Waters Reanalysis Service (SSW-RS).

For the 2023 and 2024 model runs performed in this work, we used UK-FVCOM4.3 (https://github.com/UK-FVCOM-Usergroup/uk-fvcom.git), where we implemented the OWF parameterisation, see Sect. “The underwater structure drag and mixing parameterisation”. FVCOM is coupled to the GOTM to simulate vertical turbulent mixing^18^, while horizontal mixing was parameterised in FVCOM using the Smagorinsky method with a constant horizontal diffusivity value of 0.2 m^2^s^-1^ ^27^. The atmosphere was forced by the European Centre for Medium-Range Weather Forecasts (ECMWF) dataset ERA5 (ECMWF Reanalysis 5th generation)^28^. The boundaries were forced by the Nucleus for European Modelling of the Ocean (NEMO) model of the Atlantic Margin Model 7 km (AMM7)^29,30^ and include tidal forcing from the TPXO Global Tidal Models^31^. The initial conditions were derived from the same AMM7 run. We also increased the number of sigma (terrain-following) vertical levels to 40 and improved the vertical mixing scheme after a calibration against the EN4.2.2 data^32^, which we performed to carefully check the model’s ability to reproduce the extent of mixed and stratified waters and position of the mixed layer depth (not shown). Differently from the SSW-RS run, we switched off the SST data assimilation and for lack of consistent data we closed the Baltic boundary and used a river climatology built from the 1992-2019 European-Hydrological Predictions for the Environment (E-HYPE)^33^.

### The underwater structure drag and mixing parameterisation

The sub-grid parameterisation of the underwater structure drag and wake turbulence has been described in Rennau et al.^14^, Carpenter et al.^15^, Christiansen et al.^16^. It is based on an additional quadratic friction term in the momentum equations as well as an additional production terms in the dynamic equation for TKE and its dissipation rate. In their method, the drag by a vertical cylinder perpendicular to an unstratified flow can be expressed as:

1 $$\begin{aligned} {\textbf{F}}= \frac{1}{2}\rho C_{D} A \vert {\textbf{u}}\vert {\textbf{u}} \end{aligned}$$

where *ρ * is the water density, $C_{D}$ is the structure drag coefficient, ${\textbf{u}}$ is the fluid velocity, *A* is the flow-facing area of the structure. Here we assume that the structure has a solid orthographic projection perpendicular to the dominant flow of the water, e.g. a cylinder. The restriction to cylindrical structures implies that only rotationally invariant structures are considered such that drag forces (flow deceleration) but not lift forces (flow redirection) are included. Wind turbine underwater structures are typically either monopile cylinders, tripods or four-legged (or three-legged) steel structures (jackets). Although the latter constructions are not rotationally invariant, the lift forces are believed to be small^14^. Although the parameterisation was developed for unstratified flow, Rennau et al.^14^ suggested that the basic principles of the approach also apply for stratified flow. The major argument for this is that the momentum transfer between the structure and the flow is independent of stratification, assuming that the momentum transfer into unresolved flow structures such as internal waves is negligible^14^.

To account for deceleration at grid cells containing offshore wind turbines, the drag retarding force has to be added to the hydrostatic momentum equations (with specific changes to the equations dependent on the hydrodynamic model used). The spatial average of the structure frictional force across the model grid cell has to be considered when applying the momentum sink because the horizontal resolution of the ocean model is greater than the size of structure (i.e. the structures are not directly resolved) and potentially multiple structures are present in one model cell. F in Eq 1 is divided by the mass of the water in the cell ${\rho V_{c}}$, where ${V_{c}}$ is the volume of the cell. Since the structure(s) can span multiple model vertical layers, a fraction $l_{\sigma }$ (proportional to the layer thickness) of the resistance force is applied to each model layer occupied by the structure. Thus the structure frictional force applied in one cell and one vertical layer is:

2 $$\begin{aligned} \mathbf {F_{c}}=\frac{1}{2V_{c}} N C_{D} l_{\sigma } A \vert {\textbf{u}}\vert {\textbf{u}} \end{aligned}$$

where *N* is the number of structures in the model grid cell.

For the Seagreen OWF, we modelled the 114 jackets (one turbine per model cell, *N* = 1). To calculate the flow-facing area *A*, we assumed a typical leg spacing (triangle base) of the jacket of $W_{b}$ = 32 m at the base and $W_{w}$ = 16 m at the waterline, and we assumed a fullness (the space occupied by the solid part of the jacket) of the structure of 50% ( = 0.5). This is equivalent to $A_{eq}$

by assuming 3 jacket legs ($n_{l}$) with a diameter *dl* of 3 m and adding 34% to take into account the braces too (*fb* = 0.34). These numbers are a realistic approximation for the Seagreen wind farm jackets. The flow-facing area *A* is calculated considering the water depth, *D*, in each model cell. For comparison with a cylinder structure, this would be equivalent to assume a cylinder with a diameter of 12 m.

3 $$\begin{aligned} & A = \frac{Wb+Ww}{2}D * fu \end{aligned}$$

4 $$\begin{aligned} & Aeq= (1 + fb)(nl * dl) D \end{aligned}$$

5 $$\begin{aligned} & fu =\frac{(1 + fb)(nl * dl)}{\frac{Wb+Ww}{2}} \end{aligned}$$

Taking the classical u- and v-momentum governing equations in a three-dimensional hydrostatic ocean models and adding the friction terms due to structures in the water column from Eq. 2, the following dynamic equations result:

6 $$\begin{aligned} & \frac{\partial u}{\partial t} + u \frac{\partial u}{\partial x} + v \frac{\partial u}{\partial y} + w \frac{\partial u}{\partial z} - fv = - \frac{1}{\rho _{0}}\frac{\partial p}{\partial x} + \frac{\partial }{\partial z} (K_{m} \frac{\partial u}{\partial z}) + D_{x} - F_{cx} \end{aligned}$$

7 $$\begin{aligned} & \frac{\partial v}{\partial t} + u \frac{\partial v}{\partial x} + v \frac{\partial v}{\partial y} + w \frac{\partial v}{\partial z} + fu = - \frac{1}{\rho _{0}}\frac{\partial p}{\partial y} + \frac{\partial }{\partial z} (K_{m} \frac{\partial v}{\partial z}) + D_{y} - F_{cy} \end{aligned}$$

where *u, v, w* are the three velocity components in the x, y, and z directions; $D_{x}, D_{y}$ are the horizontal momentum diffusivity terms in the x and y directions; $K_{m}$ is the vertical eddy viscosity coefficient; *p* is pressure; and *f* is the Coriolis parameter. $F_{cx}$ and $F_{cy}$ are the added momentum sink terms due to the presence of the structures.

FVCOM is a 3-D unstructured-grid coastal ocean model that simulates water surface elevation, velocity, temperature, salinity. The unstructured grid and finite volume approach employed in the model provides geometric flexibility and computational efficiency that is well suited to simulating the effect of underwater structures on a flow field at a fine scale within in a large domain. FVCOM uses unstructured triangular cells in the horizontal plane and a sigma-stretched coordinate system in the vertical. A mode splitting approach is used in the numerical scheme to solve the depth-averaged 2-D barotropic external mode and 3-D baroclinic internal mode equations. The integrated form Eqs. 6 and 7 for the 3-D internal mode become:

8 $$\begin{aligned} & \frac{\partial (A_{c}\Delta D u)}{\partial t} + R_{u} - fA_{c}\Delta Dv = -\frac{1}{2} N C_{D} l_{\sigma } A \sqrt{(u^{2}+v^{2})} u \end{aligned}$$

9 $$\begin{aligned} & \frac{\partial (A_{c}\Delta D v)}{\partial t} + R_{v} + fA_{c}\Delta Du = -\frac{1}{2} N C_{D} l_{\sigma } A \sqrt{(u^{2}+v^{2})} v \end{aligned}$$

where $A_{c}$ is the surface horizontal area of the triangular cell and *Δ D* is the sigma -layer thickness ($V_{c}$ the volume of the model cell in Eq. 2 is equal to $A_{C}\Delta D$). $R_{u}$ and $R_{v}$ are all the remaining momentum terms including advection, diffusion, and pressure gradient. The right-hand side of Eqs. 8 and 9 is the momentum sink term of the wind turbine underwater structures defined in Eq. 2.  

Correspondingly, the integrated form for the 2-D external mode of Eqs. 6 and 7 becomes:

10 $$\begin{aligned} & \frac{\partial ( A_{c} D {\bar{u}} )}{\partial t} + R_{{\bar{u}}} - fA_{c} D{\bar{v}} = -\frac{1}{2} \sum _{\sigma =1}^{\sigma =n_{L}} C_{D} N A l_{\sigma }\sqrt{(u^{2}+v^{2})} u \end{aligned}$$

11 $$\begin{aligned} & \frac{\partial ( A_{c} D {\bar{v}} )}{\partial t} + R_{{\bar{v}}} + fA_{c} D{\bar{u}} = -\frac{1}{2} \sum _{\sigma =1}^{\sigma =n_{L}} C_{D} N A l_{\sigma }\sqrt{(u^{2}+v^{2})} v \end{aligned}$$

where ${\bar{u}}$ and ${\bar{v}}$ are the vertically integrated velocity in the x and y directions, respectively, *D* is the total depth, *σ* is the vertical sigma-layer index and $n_{L}$ is the total number of vertical sigma layers.

In order to account for the production of turbulence due the presence of the structures, the driving assumption is that the turbulence produced by the underwater structures is equal to the power lost to drag, $P_{d}$ for each layer is:

12 $$\begin{aligned} {P_{d}}= \frac{1}{2 V_{c}} N C_{D} l_{\sigma } A (u^{2}+v^{2})^{3/2} \end{aligned}$$

$P_{d}$ is then included as further production term in the TKE equation and as a source term into the dissipation rate as well, scaled by an empirical parameter $c_{4}$.

In this work we use GOTM^18^, coupled with FVCOM. GOTM is a one-dimensional stand-alone ocean model which has been developed specifically to be coupled with 3D ocean models and provide higher order turbulence models for use when solving hydrodynamic model equations. We chose the *k - ε * turbulent closure scheme, which in its form suggested by Rodi^34^ has been implemented in GOTM. In this study, the TKE and its dissipation rate calculated by means of the *k - ε * two-equation model have been modified with the introduction of turbulence due to the presence of the underwater structures. $P_{d}$ was added as a production term in the TKE equation (Eq. 13) and as a source term in the dissipation equation scaled by an empirical parameter $c_{4}$ (Eq. 14). Using this method, the effects are simulated through the water column:

13 $$\begin{aligned} & \frac{\partial k}{\partial t} - \frac{\partial }{\partial z} \left( \frac{\nu _{t}}{s_{k}}\frac{\partial k}{\partial z}\right) = P + P_{d} + B - \epsilon \end{aligned}$$

14 $$\begin{aligned} \frac{\partial \epsilon }{\partial t} - \frac{\partial }{\partial z} \left( \frac{\nu _{t}}{s_{\epsilon }}\frac{\partial \epsilon }{\partial z}\right) = \frac{\epsilon }{k}(c_{1}P + c_{4}P_{d} + c_{3}B -c2\epsilon ) & \end{aligned}$$

where *P* is the turbulent shear production and *B* is the turbulent buoyancy production, $\nu _{t}$ is the vertical eddy viscosity. Other constant empirical parameters are the Schmidt numbers, $s_{k}$ and $s_{\epsilon }$ and the model-specific weighting parameters $c_{1}$, $c_{2}$, $c_{3}$ and $c_{4}$ for the source and sink terms in the *ε * equation. $c_{4}$ is the scaling parameter associated with dissipation of turbulence due to the OWF structures.

## Supplementary Information

Supplementary Information.

## Data Availability

PELAgIO 2023/2024 surveys, that support the findings of this study, are available from the British Oceanographic Data Centre (BODC).

## Code availability

The FVCOM code is available on GitHub in the UK FVCOM Users’ repository at https://github.com/UK-FVCOM-Usergroup/uk-fvcom/tree/FVCOM-EnergyVeg_GOTM, commit e4c6558662a363ca94a40ac6d00c092a49e6aa2e. The modifications to the GOTM are available on Zenodo at https://doi.org/10.5281/zenodo.22646209.
