# Supplementary material for: Environmental controls on the detectability of hydrodynamic changes induced by offshore wind farm structures
Source: Sci Rep. 2026 Oct 7;16:30218. doi: 10.1038/s41598-026-71585-0 (PMC13645625; doi:10.1038/s41598-026-71585-0)
Supplement: Supplementary file 1 [file 41598_2026_71585_MOESM1_ESM.pdf]

Supplementary Material - Environmental controls on the detectability of hydrodynamic changes induced by offshore wind farm structures

Michela De Dominicis[1*], Rory O'Hara Murray[2], Alejandro Gallego[2], Charlotte Williams[1], Beth Scott[3]

[1*]National Oceanography Centre, Liverpool, UK.
[2]Scottish Government Marine Directorate, Aberdeen, UK.
[3]University of Aberdeen, Aberdeen, UK.

*Corresponding author(s). E-mail(s): micdom@noc.ac.uk;

S1 Monthly variability of hydrodynamic changes induced by offshore wind farm structures

During May 2023 and May 2024, Bottom Temperature (BT) changes induced by the Seagreen Offshore Wind Farm (OWF) structures were not present (Figure S1b,d), as was the case for Sea Surface Temperature (SST) (main paper, Figure 5). During summer months BT changes are usually around 0.1°C, reaching peaks of 0.2° in 2023 (Figure S1f,j,n), while consistently around 0.1°C in 2024 (Figure S1h,l,p).

OWF-induced changes in Turbulent Kinetic Energy (TKE) show the largest increases in May 2023 and May, August 2024 (Figure S2b,d,p). Throughout the summer months of both years, TKE consistently increases due to the presence of the Seagreen OWF. However, the magnitude of these changes is generally larger in summer 2024 (Figure S2h,l,p), with the strongest enhancement occurring in August 2024 (Figure S2p).

Despite the larger OWF-induced increases in mixing in 2024, the OWF-induced changes in Potential Energy Anomaly (PEA) are greater during the summer months of 2023. The most pronounced reductions occur in June and August 2023, with decreases reaching up to –4 J m^{-3} in August 2023 (Figure S3n). In 2024, changes in PEA are negligible (Figure S3h,l,p) because the water column was not stratified during that summer (Figure S3g,k,o).

Drag exerted by the underwater structures reduces surface current speeds by up to 0.02 m/s (5%), with the largest reductions occurring during May 2023 and May 2024 (Figure S4b,d). The reduction is primarily confined to the upper water column, while an increase in current speed is also evident at mid-depth, both years and every month (Figure S5), while the wake is not discernible at the seabed bottom (Figure S6).

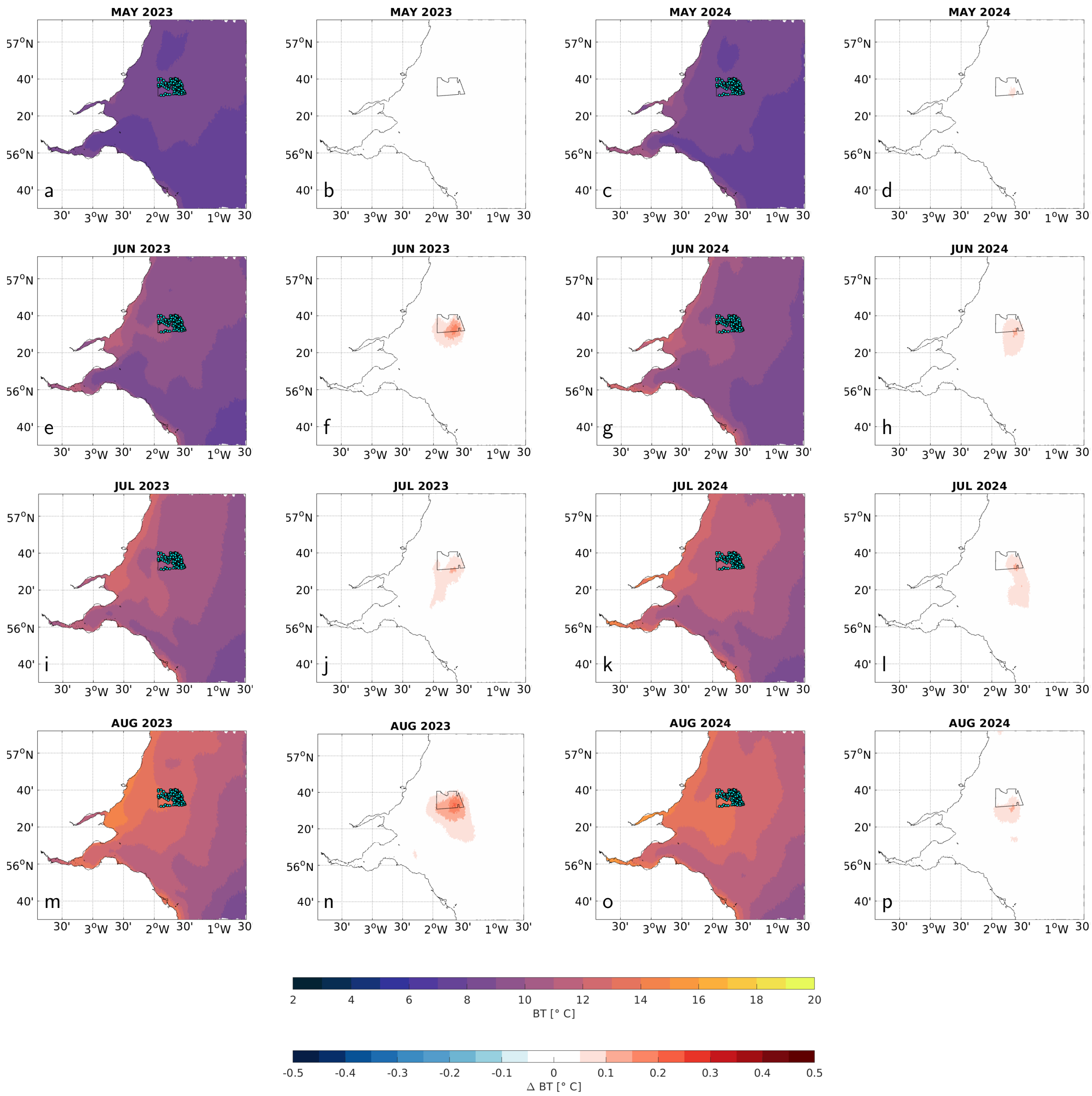

Fig. S1 Monthly average BT and changes due to the Seagreen wind farm structure wake in 2023 and 2024 ($C_D = 0.63$, $c_4 = 0.6$). BT changes are calculated as the difference between the monthly average simulation including the OWF and the baseline simulation without the OWF. Panels show: a) BT May 2023; b) BT changes May 2023; c) BT May 2024; d) BT changes May 2024; e) BT June 2023; f) BT changes June 2023; g) BT June 2024; h) BT changes June 2024; i) BT July 2023; j) BT changes July 2023; k) BT July 2024; l) BT changes July 2024; m) BT August 2023; n) BT changes August 2023; o) BT August 2024; p) BT changes August 2024. The polygon outlines the perimeter of the Seagreen wind farm and the light blue dots are the locations of the wind turbines.

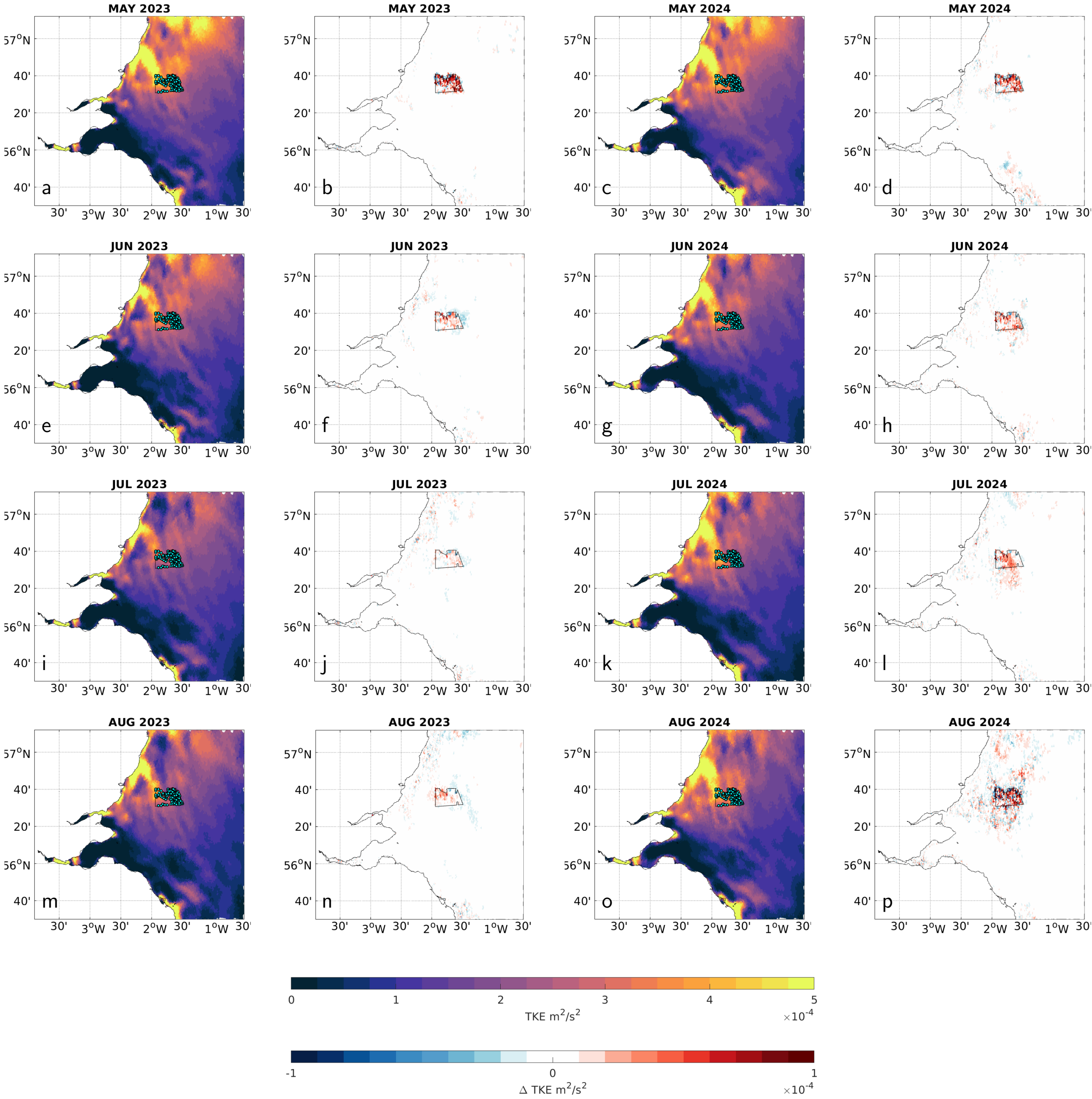

Fig. S2 Monthly average mid-water TKE and TKE changes due to the Seagreen wind farm structure wake in 2023 and 2024 ($C_D = 0.63$, $c_4 = 0.6$). TKE changes are calculated as the difference between the monthly average simulation including the OWF and the baseline simulation without the OWF. Panels show: a) TKE May 2023; b) TKE changes May 2023; c) TKE May 2024; d) TKE changes May 2024; e) TKE June 2023; f) TKE changes June 2023; g) TKE June 2024; h) TKE changes June 2024; i) TKE July 2023; j) TKE changes July 2023; k) TKE July 2024; l) TKE changes July 2024; m) TKE August 2023; n) TKE changes August 2023; o) TKE August 2024; p) TKE changes August 2024. The polygon outlines the perimeter of the Seagreen wind farm and the light blue dots are the locations of the wind turbines.

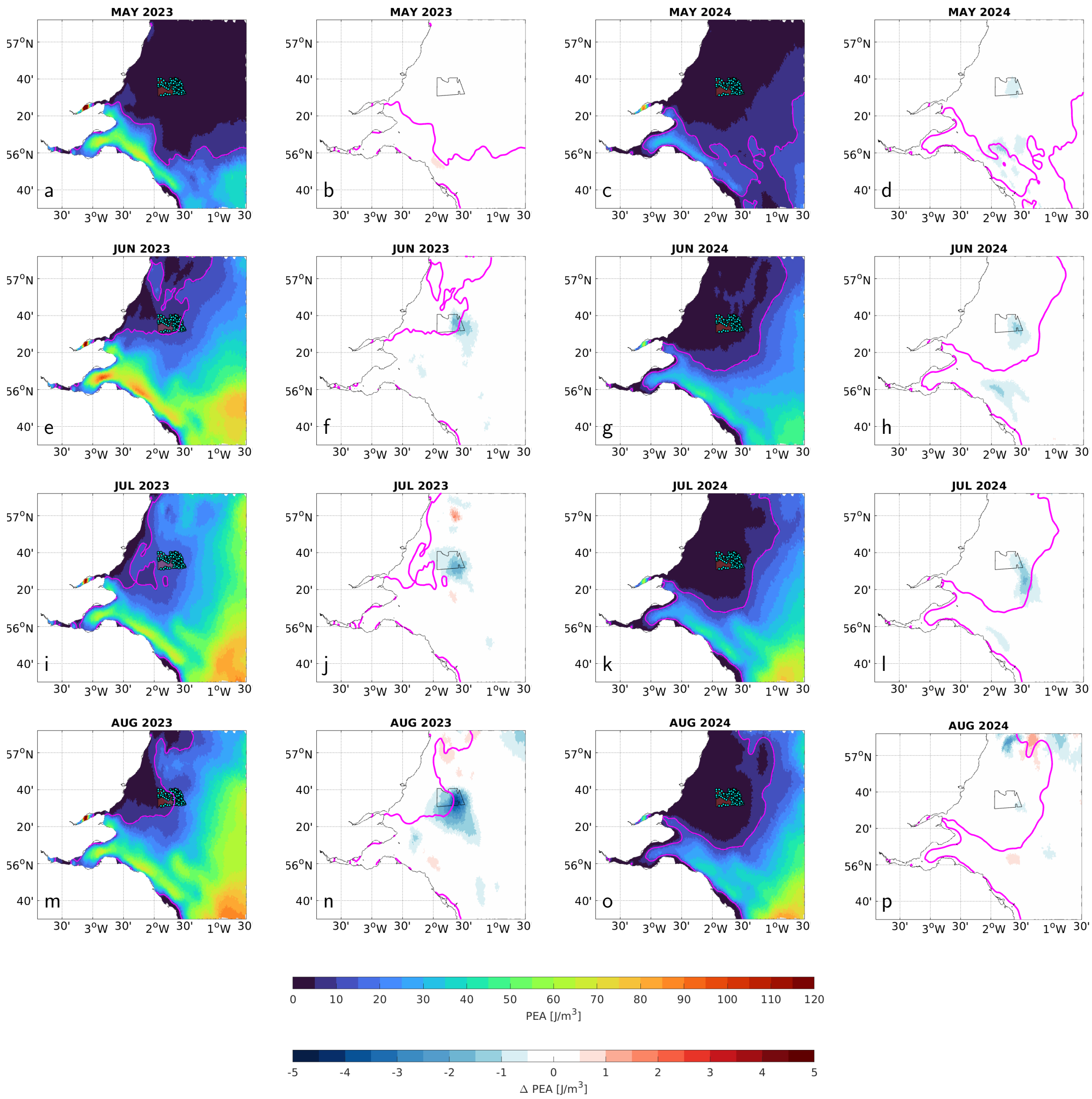

Fig. S3 Monthly average PEA and changes due to the Seagreen wind farm structure wake in 2023 and 2024 ($C_D = 0.63$, $c_4 = 0.6$). PEA changes are calculated as the difference between the monthly average PEA from the simulation including the OWF and the baseline simulation without the OWF. Panels show: a) PEA May 2023; b) PEA changes May 2023; c) PEA May 2024; d) PEA changes May 2024; e) PEA June 2023; f) PEA changes June 2023; g) PEA June 2024; h) PEA changes June 2024; i) PEA July 2023; j) PEA changes July 2023; k) PEA July 2024; l) PEA changes July 2024; m) PEA August 2023; n) PEA changes August 2023; o) PEA August 2024; p) PEA changes August 2024. The polygon outlines the perimeter of the Seagreen wind farm and the light blue dots are the locations of the wind turbines.

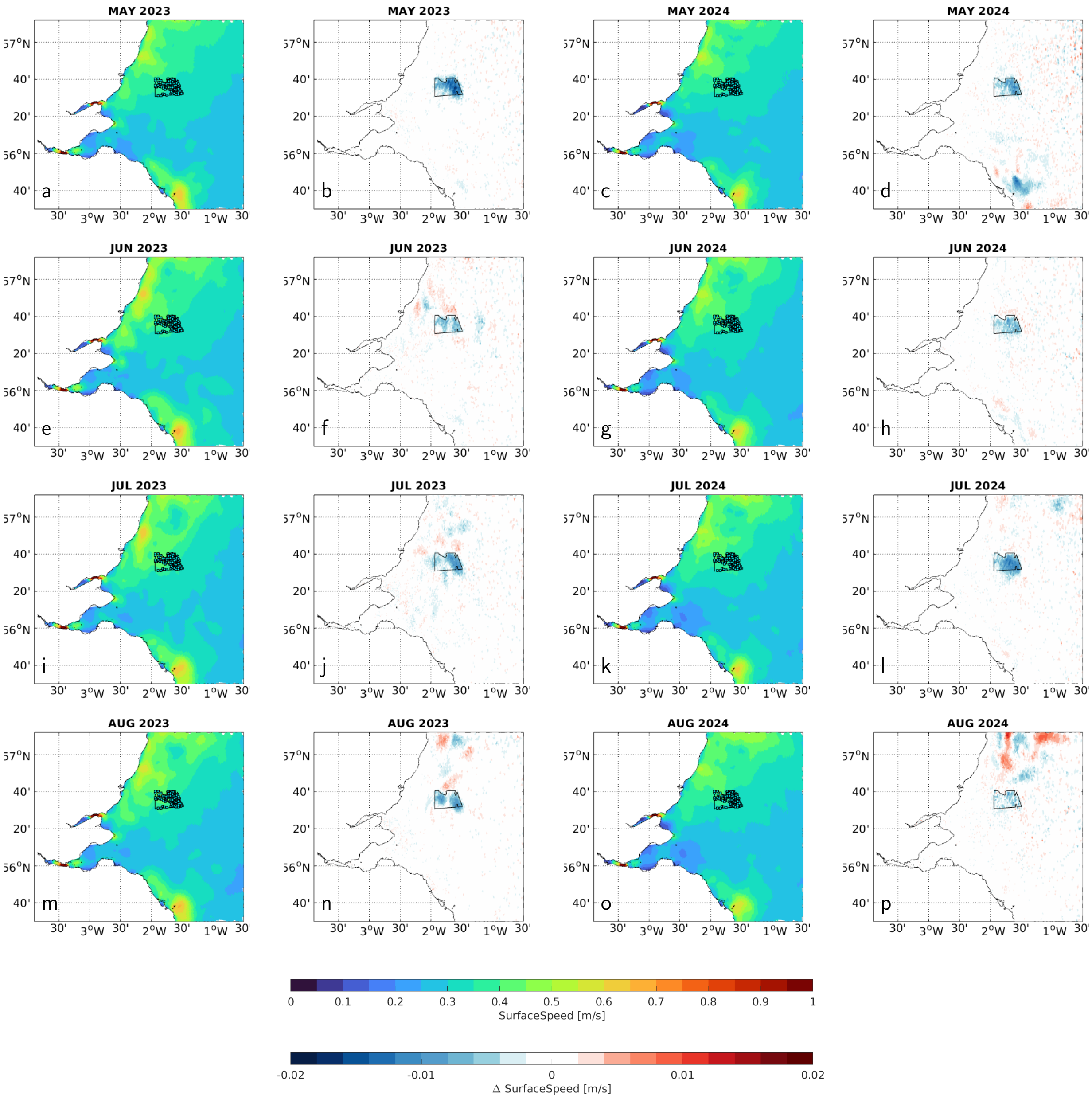

Fig. S4 Monthly average Surface Current Speed and changes due to the Seagreen wind farm structure wake in 2023 and 2024 ($C_D = 0.63$, $c_4 = 0.6$). Surface Current Speed changes are calculated as the difference between the monthly average Surface Current Speed from the simulation including the OWF and the baseline simulation without the OWF. Panels show: a) Surface Current Speed May 2023; b) Surface Current Speed changes May 2023; c) Surface Current Speed May 2024; d) Surface Current Speed changes May 2024; e) Surface Current Speed June 2023; f) Surface Current Speed changes June 2023; g) Surface Current Speed June 2024; h) Surface Current Speed changes June 2024; i) Surface Current Speed July 2023; j) Surface Current Speed changes July 2023; k) Surface Current Speed July 2024; l) Surface Current Speed changes July 2024; m) Surface Current Speed August 2023; n) Surface Current Speed changes August 2023; o) Surface Current Speed August 2024; p) Surface Current Speed changes August 2024. The polygon outlines the perimeter of the Seagreen wind farm and the light blue dots are the locations of the wind turbines.

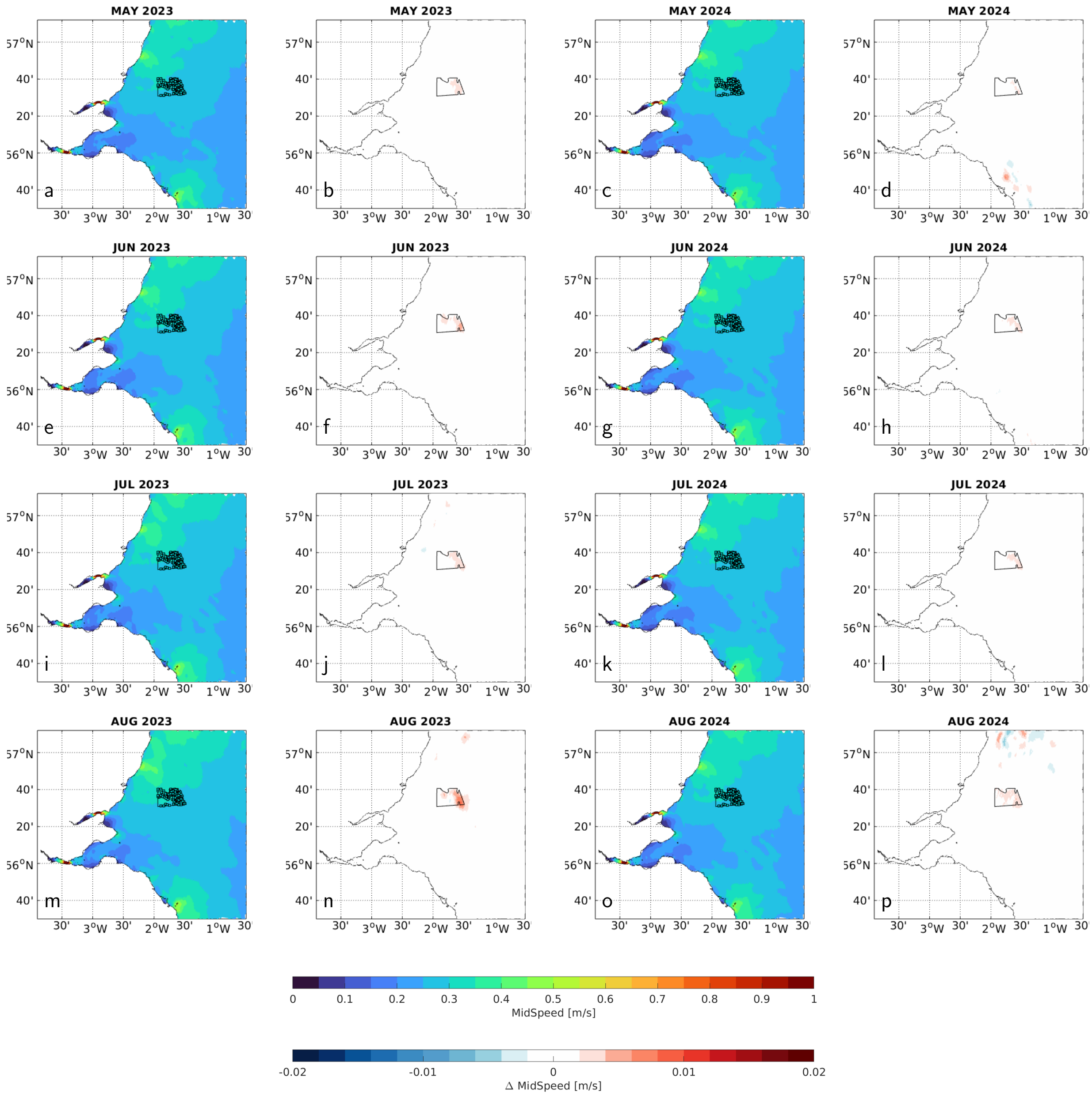

Fig. S5 Monthly average Mid-column Speed and changes due to the Seagreen wind farm structure wake in 2023 and 2024 ($C_D = 0.63$, $c_4 = 0.6$). Mid-column Current Speed changes are calculated as the difference between the monthly average Mid-column Current Speed from the simulation including the OWF and the baseline simulation without the OWF. Panels show: a) Mid-column Current Speed May 2023; b) Mid-column Current Speed changes May 2023; c) Mid-column Current Speed May 2024; d) Mid-column Current Speed changes May 2024; e) Mid-column Current Speed June 2023; f) Mid-column Current Speed changes June 2023; g) Mid-column Current Speed June 2024; h) Mid-column Current Speed changes June 2024; i) Mid-column Current Speed July 2023; j) Mid-column Current Speed changes July 2023; k) Mid-column Current Speed July 2024; l) Mid-column Current Speed changes July 2024; m) Mid-column Current Speed August 2023; n) Mid-column Current Speed changes August 2023; o) Mid-column Current Speed August 2024; p) Mid-column Current Speed changes August 2024. The polygon outlines the perimeter of the Seagreen wind farm and the light blue dots are the locations of the wind turbines.

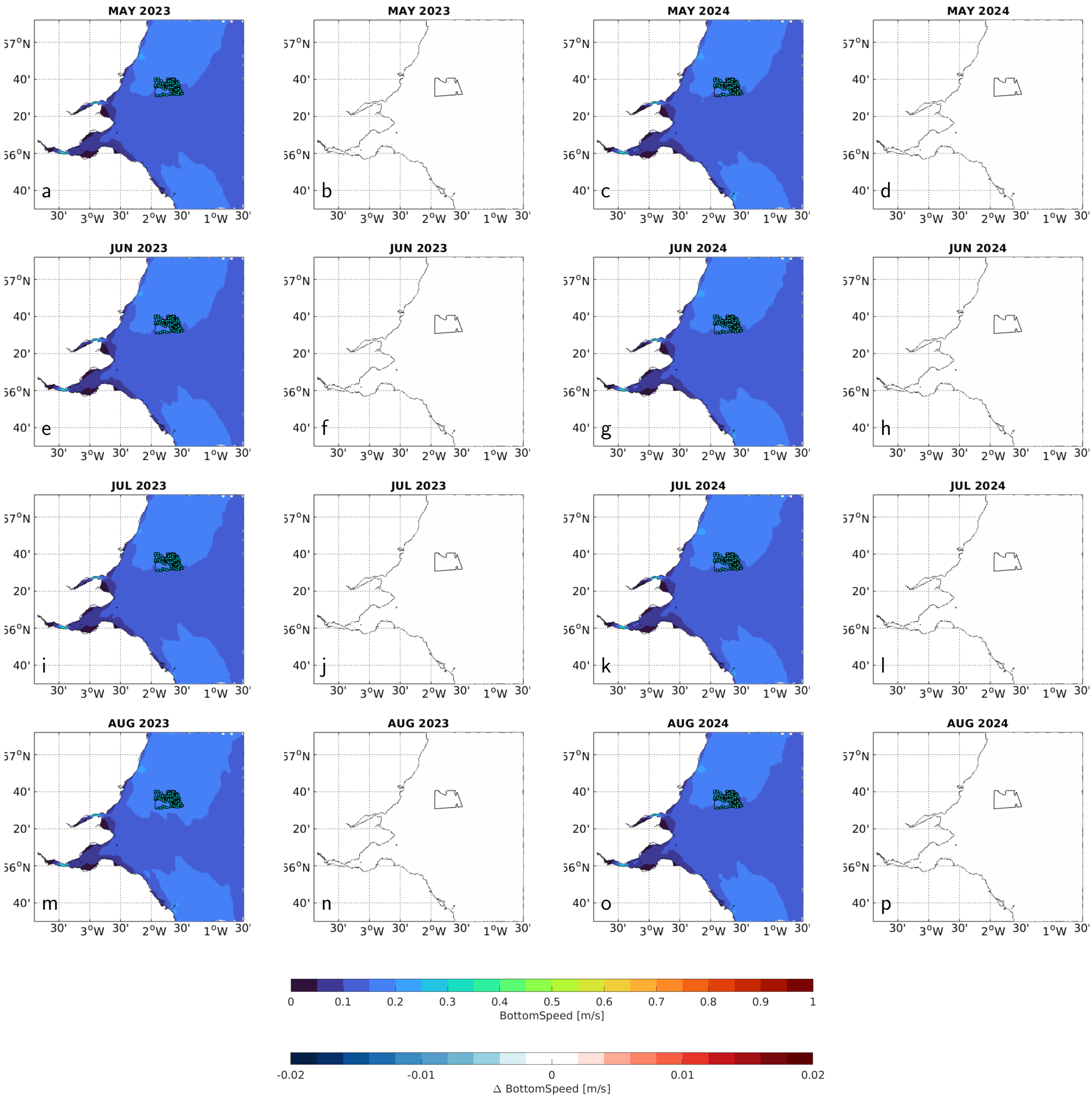

Fig. S6 Monthly average Bottom Speed and changes due to the Seagreen wind farm structure wake in 2023 and 2024 ($C_D = 0.63$, $c_4 = 0.6$). Bottom Current Speed changes are calculated as the difference between the monthly average Bottom Current Speed from the simulation including the OWF and the baseline simulation without the OWF. Panels show: a) Bottom Current Speed May 2023; b) Bottom Current Speed changes May 2023; c) Bottom Current Speed May 2024; d) Bottom Current Speed changes May 2024; e) Bottom Current Speed June 2023; f) Bottom Current Speed changes June 2023; g) Bottom Current Speed June 2024; h) Bottom Current Speed changes June 2024; i) Bottom Current Speed July 2023; j) Bottom Current Speed changes July 2023; k) Bottom Current Speed July 2024; l) Bottom Current Speed changes July 2024; m) Bottom Current Speed August 2023; n) Bottom Current Speed changes August 2023; o) Bottom Current Speed August 2024; p) Bottom Current Speed changes August 2024. The polygon outlines the perimeter of the Seagreen wind farm and the light blue dots are the locations of the wind turbines.

S2 Model–observation comparison using CTD profiles

Here, using Conductivity Temperature Depth (CTD) observational data we perform a validation of the choice of drag coefficient C_D and turbulence dissipation scaling parameter c_4 (see main paper, Section "The underwater structure drag and mixing parameterisation").

Experimental studies show that the drag coefficient for a cylinder in an unstratified fluid can range widely, typically between $C_D = 0.2$ and $C_D = 1.3$ [1, 2], depending on factors such as Reynold's number, turbulence intensity in the approaching flow and surface roughness. Because these conditions vary with structure design, location, and local hydrodynamics, using a fixed drag coefficient may systematically over- or underestimate the frictional processes. To retain and quantify this uncertainty in our analysis, we use three estimates of C_D that span the expected range, $C_D = 0.35$, 0.63, 1.0, which we refer to as the Low, Medium and High drag cases, respectively (see Table S1). Those values were also used in previous studies [3–5]. Here, we compare model results with observational data to constrain the appropriate drag coefficient and assess whether variations in C_D lead to fundamental differences in the predicted impacts. Without this validation, the reliability of model-based estimates would remain limited. We test also different values of c_4, the physical meaning of c_4 could be identified as connected to the structure-induced mixing efficiency (see main paper, Section "The underwater structure drag and mixing parameterisation" for the full set of equations where c_4 is used). Rennau et al. [4] derived the c_4 parameter by calibrating an extended $k-\epsilon$ turbulence closure model by comparing LES simulations of stratified flow past 10 m diameter cylinders and determined an upper limit value of $c_4 = 1.75$ and suggested $c_4 = 0.6$ for strong mixing scenarios and $c_4 = 1.4$ for weak mixing scenarios, in which the dissipation response to OWF-induced turbulence production is enhanced. We tested $c_4 = 0.6$ and 1.4 and also $c_4 = 0$, for which the dissipation associated with OWF-induced turbulence production is excluded, resulting in enhanced mixing (see Table S1).

The model reproduced the observed 2023 CTD temperature profiles with similar skill across all simulations (Table S1). The mean RMSE across the 89 CTD profiles ranged from 0.135 to 0.137°C, with the lowest value obtained for the high-drag simulation ($C_D = 1.0$, $c_4 = 0.6$). Temperature was consistently underestimated, with biases approximately -0.02°C. Differences in RMSE and bias among the sensitivity experiments are too small to be statistically significant, indicating that, due to environmental conditions of May 2023, the tested structure-drag and turbulence-dissipation parameters had little influence on the overall model–observation agreement.

In Figure S7, we show a comparison of CTD data collected between 6 May 2023 and 11 May 2023. Out of a total of 89 CTD profiles, we selected seven representative casts to illustrate the progression of water column conditions from the beginning of the survey, when the water column was still partially mixed, to the end of the survey, when stratification had developed. Selected CTD profiles span the entire survey period and were taken in the vicinity of the southeastern corner of the Seagreen wind farm (CTD profile 4, 22, 38, 56, and 74, see locations in Figure S7), where the effects of the underwater structures on water properties were the largest. In particular, CTD profile 56 and 74 were the closest profiles to the wind turbines, collected within the Seagreen wind farm itself. Additionally, two CTD profiles (85 and 89, see locations in Figure

Table S1 Numerical experiments and model–CTD profile comparison. The baseline simulation excludes structure-induced drag ($C_D = 0$) and additional turbulence production. Sensitivity experiments vary the drag coefficient (C_D) or the turbulence dissipation scaling parameter (c_4). EM and WM denote enhanced- and weak-mixing scenarios, respectively. RMSE and bias are calculated for the 89 and 77 CTD profiles collected in May 2023 and 2024, respectively, using the nearest model level to each observation. RMSE and bias are averaged over the water column.

Experiment	C_D	c_4	RMSE (°C)	Bias (°C)
May 2023				
Baseline	0	0	0.1371	-0.0233
Low drag	0.35	0.6	0.1360	-0.0256
Medium drag	0.63	0.6	0.1362	-0.0260
High drag	1.00	0.6	0.1352	-0.0261
Medium drag EM	0.63	0	0.1366	-0.0261
Medium drag WM	0.63	1.4	0.1363	-0.0260
May 2024				
Baseline	0	0	0.2439	-0.0189
Low drag	0.35	0.6	0.2442	-0.0189
Medium drag	0.63	0.6	0.2467	-0.0192
High drag	1.00	0.6	0.2474	-0.0184

S7) were selected from the end of the survey, when the water column was stratified, as these conditions exhibit greater sensitivity to C_D and c_4 parameters, even if the CTD were further away from the Seagreen wind farm.

Some differences between the model and the observations are evident: the model stratifies earlier than observed, there is a small bottom temperature bias, and the mixed layer depth is occasionally underestimated. However, what is striking is that, at least for May 2023, the effects of varying C_D and c_4 parameters are relatively small. While differences are visible, they do not produce substantially different results, making it challenging to determine which parameter set best reproduces the observations using this particular set of CTD data. In some cases, such as CTD profile 56 (see Figure S7), including the OWF underwater structure parameterisation improves the match with observations, by decreasing the modelled top temperature of about -0.2°C, although there is little sensitivity to the choice of parameters. For the CTD profiles 89 and 74 (see Figure S7), increasing the drag coefficient (C_D) to 1 results in a worse agreement with observations. Only for the CTD profile 89, the model shows a modest sensitivity to C_D, with changes in the surface temperature of approximately 0.2°C between $C_D = 1$ and $C_D = 0.35$. The sensitivity to the c_4 parameters is negligible for all profiles. The limited sensitivity to model parameters (C_D and c_4) can partly be attributed to the fact that the CTD deployment time and location were not optimal to detect the changes induced by the OWF underwater structures. A larger sensitivity would be expected under stronger stratified conditions and higher tidal velocities.

Indeed, CTD profile 89 (see Figure S7), collected at the end of the May 2023 survey when these conditions were present, exhibits the largest sensitivity. Surface temperature changes induced by the OWF underwater structures and the sensitivity to model parameters can reach up to ±0.1-0.2°C, but these changes are comparable to the model's inherent error (mean RMSE of 0.13°C for May 2023; Table S1).

The model reproduced the 2024 CTD temperature profiles with higher errors than in 2023 across all simulations (Table S1). The mean RMSE across the 77 CTD profiles ranged from 0.244 to 0.247°C, while the mean bias is approximately -0.02°C. Again, the differences among the sensitivity experiments were small and not statistically significant, indicating that variations in structure-induced drag had little influence on the overall model–observation agreement.

Next, we show selected seven CTD profiles from the 77 collected during the Spring 2024 survey (3–19 May 2024). Three of these were located within the Seagreen wind farm (CTD profile 17, 18, and 56, see locations in Figure S8), while the remaining profiles were taken very close to the southeastern corner of the OWF (CTD profile 42, 50, 63, and 73, see locations in Figure S8). As in 2023, the 2024 results show very limited sensitivity to the C_D (we did not test the sensitivity to the c_4 parameter in the 2024 simulations, given the negligible sensitivity detected in the 2023 simulations). For CTD profile 18 (see Figure S8), the maximum reduction in the top temperature is obtained with $C_D = 1$, but the magnitude of the reduction is only approximately 0.1°C and leads to a poorer agreement with the observations. For CTD profile 42 (see Figure S8), increasing C_D to 1 results in the top temperature reduction of approximately 0.2°C, whereas a smaller reduction (0.1°C) is obtained with C_D=0.63, which appears to provide a better match with the CTD data.

However, the surface temperature reduction due to the presence of the OWF structures and the sensitivity to the model parameters are below the mean RMSE (approximately 0.2°C in May 2024 see Table S1) making it difficult to clearly distinguish parameter sensitivity when it is of the same order of magnitude as the model error.

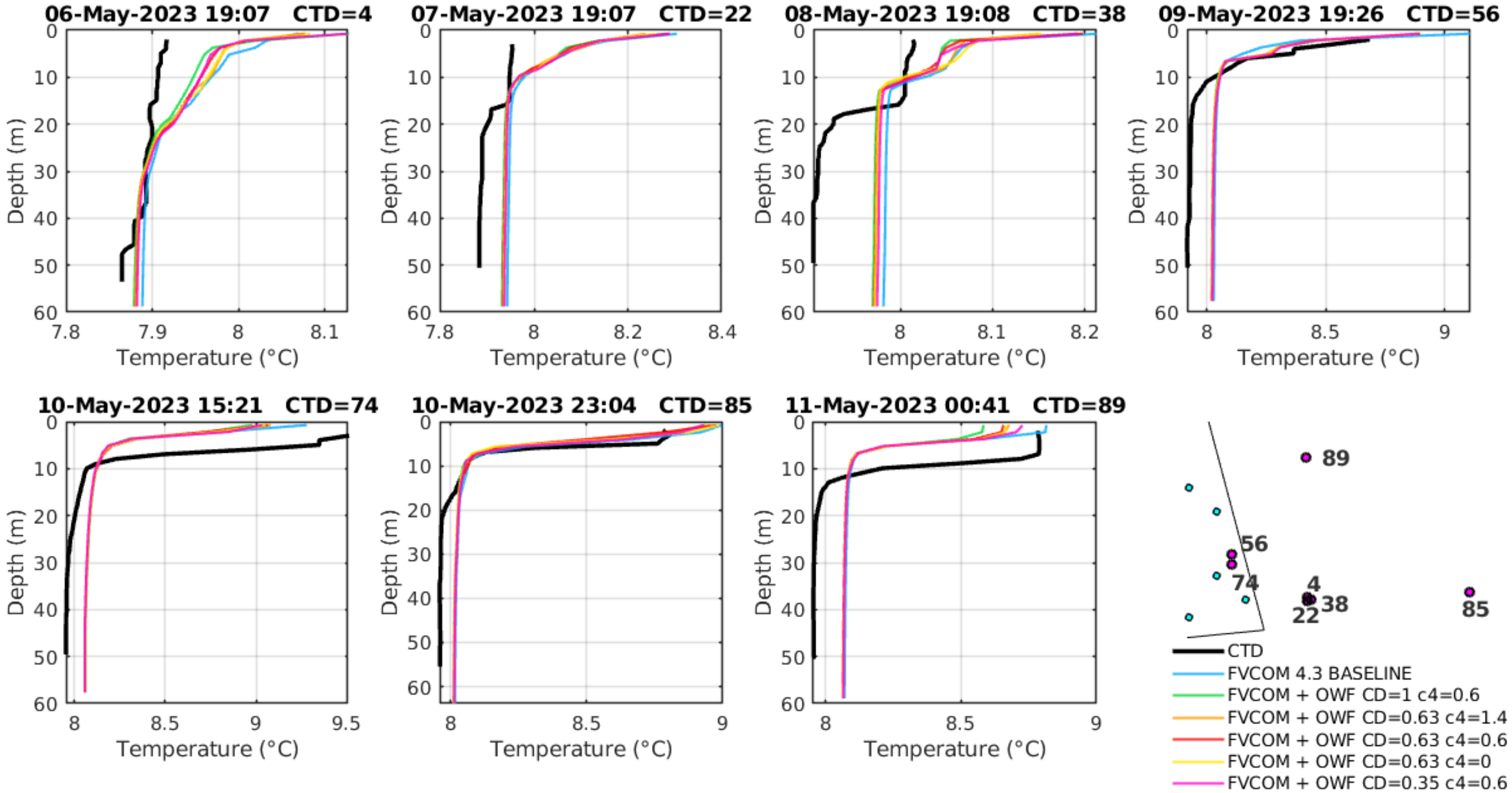

Fig. S7 Comparison of selected CTD temperature profiles (black) and model simulations (coloured lines) between 6 May 2023 and 11 May 2023. Seven representative CTD profiles were chosen from a total of 89 profiles. Model simulations include variations in drag coefficient (C_D) and the c_4 parameter. The locations of the selected CTD deployment (pink dots) are referenced to the south-east corner of Seagreen wind farm and turbine locations (blue dots). Varying axis limits are used to better illustrate the spread of the simulations, which would otherwise be difficult to visualize.

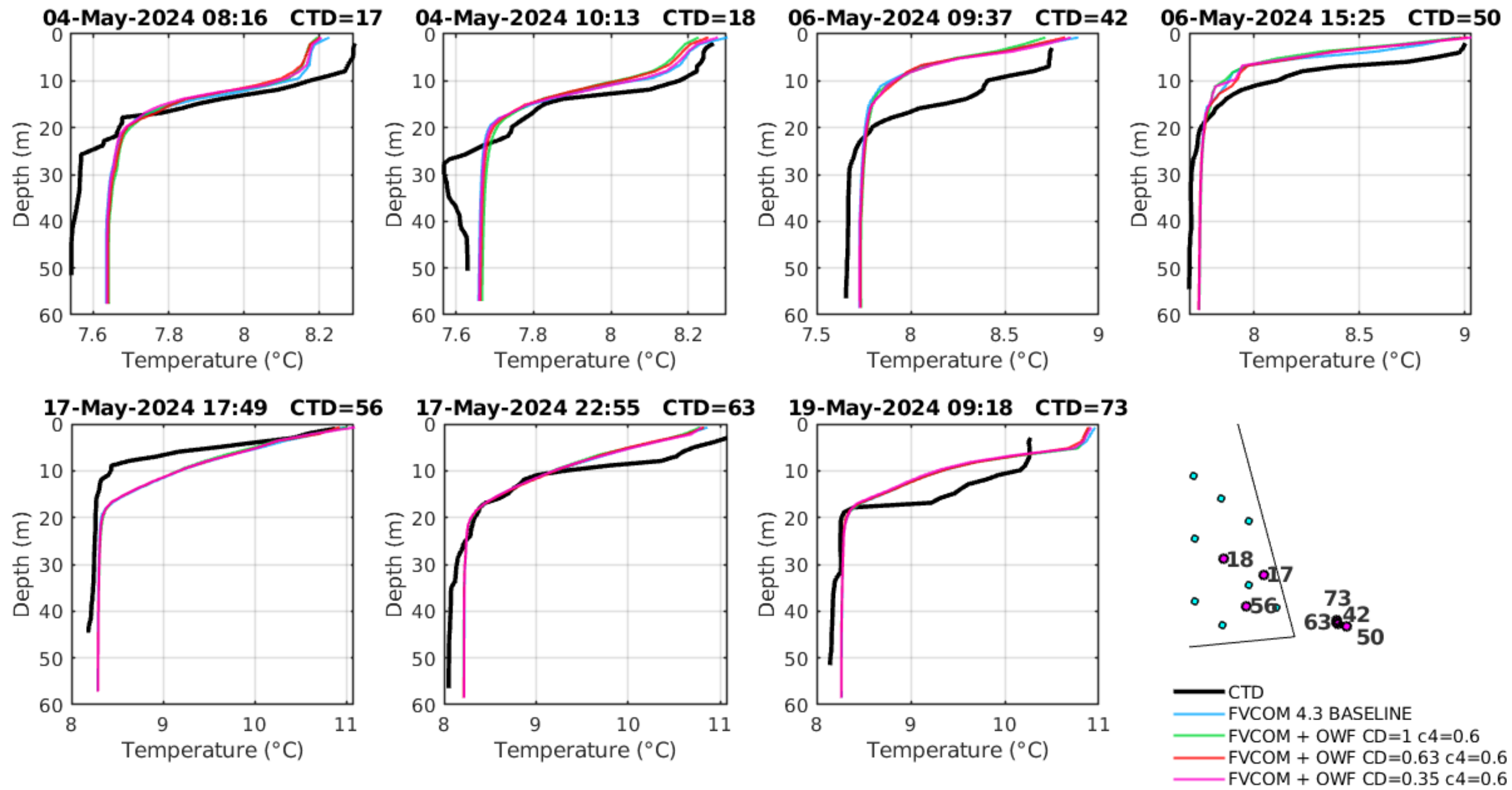

Fig. S8 Comparison of selected CTD temperature profiles (black) and model simulations (coloured lines) between 3 May 2024 and 19 May 2024. Seven representative CTD profiles were chosen from a total of 77 profiles. Model simulations include variations in drag coefficient (C_D). The locations of the selected CTD deployment (pink dots) are referenced to the south-east corner of Seagreen wind farm and turbine locations (blue dots). Varying axis limits are used to better illustrate the spread of the simulations, which would otherwise be difficult to visualize.

References

[1] Shih, W., Wang, C., Coles, D., Roshko, A.: Experiments on flow past rough circular cylinders at large reynolds numbers. Journal of Wind Engineering and Industrial Aerodynamics **49**(1-3), 351–368 (1993)

[2] Sumer, B.M., Fredsøe, J.: Hydrodynamics Around Cylindrical Structures vol. 26, (2006)

[3] Carpenter, J.R., Merckelbach, L., Callies, U., Clark, S., Gaslikova, L., Baschek, B.: Potential impacts of offshore wind farms on north sea stratification. PloS one **11**(8), 0160830 (2016)

[4] Rennau, H., Schimmels, S., Burchard, H.: On the effect of structure-induced resistance and mixing on inflows into the baltic sea: a numerical model study. Coastal Engineering **60**, 53–68 (2012)

[5] Christiansen, N., Carpenter, J.R., Daewel, U., Suzuki, N., Schrum, C.: The large-scale impact of anthropogenic mixing by offshore wind turbine foundations in the shallow North Sea. Frontiers in Marine Science **10**, 838 (2023)
